# A four-DNA methylation signature as a novel prognostic biomarker for survival of patients with gastric cancer

**DOI:** 10.1186/s12935-020-1156-8

**Published:** 2020-03-20

**Authors:** Chunmei Li, Ya Zheng, Ke Pu, Da Zhao, Yuping Wang, Quanlin Guan, Yongning Zhou

**Affiliations:** 1 Key Laboratory for Gastrointestinal Diseases, Gansu Province, The First Hospital of Lanzhou University, Lanzhou, China; 2 Department of Oncology, The First Hospital of Lanzhou University, Lanzhou, China; 3 Department of Gastroenterology, The First Hospital of Lanzhou University, Lanzhou, China; 4 Department of Oncology Surgery, The First Hospital of Lanzhou University, Lanzhou, China

**Keywords:** DNA methylation, Gastric cancer, Prognosis, Biomarker

## Abstract

**Background:**

Gastric cancer (GC) is the fifth most frequently diagnosed cancer and the third leading cause of cancer-related mortality. Lack of prognostic indicators for patient survival hinders GC treatment and survival.

**Methods and results:**

Methylation profile data of patients with GC obtained from The Cancer Genome Atlas (TCGA) database were analyzed to identify methylation sites as biomarkers for GC prognosis. The cohort was divided into training and validation sets. Univariate Cox, LASSO regression,and multivariate Cox analyses revealed a close correlation of a four-DNA methylation signature as a risk score model with the overall survival of patients with GC. The survival between high-risk and low-risk score patients with GC was significantly different. Analyses of receiver operating characteristics revealed a high prognostic accuracy of the four-DNA methylation signature in patients with GC. The subgroup analysis indicated that the accuracy included that for anatomical region, histologic grade, TNM stage, pathological stage, and sex. The GC prognosis based on the four-DNA methylation signature was more precise than that based on known biomarkers.

**Conclusions:**

The four-DNA methylation signature could serve as a novel independent prognostic factor that could be an important tool to predict the prognostic outcome of GC patients. This potential must be verified in a large-scale population cohort study and through basic research studies.

## Background

Gastric cancer (GC) is the fifth most frequently diagnosed cancer and the third leading cause of cancer-related mortality [1]. According to the 2018 global cancer report, more than one million new cases of GC were reported, and 783,000 deaths were attributed to GC [1]. While the prevalence of stomach cancer has decreased in most Western countries, it remains one of the most common causes of cancer-related deaths in Asia [1–3]. Patients are typically asymptomatic during early stages, which can delay diagnosis. Diagnosis at an advanced stage of the disease is associated with poor overall survival (OS). Current treatments for GC include surgical resection, chemotherapy, targeted therapy, and radiotherapy [3, 4]. The tumor, node, metastasis (TNM) stage is vital for defining patient prognosis and the choice of therapeutic strategies [5–7]. Accurate staging and treatment following the guidelines could differentiate OS for the patients with the same TNM stage [8, 9]. A precise predictive tool for the prognosis of patients with GC and the choice of individual treatment are hot topics.

An increasing number of studies on the pathogenesis of tumors are directed towards epigenetics. The spatial conformation of DNA and its transcriptional activity are affected by epigenetic changes, which are inherited and reversible. Epigenetics includes abnormal DNA methylation, editing of coding RNA and non-coding RNA, and histone modification [10, 11]. DNA methylation annotates genomic regions, which determines when and how information is read, as well as transcription control [12]. The DNA in tumor cells is more readily methylation compared to that in normal cells [13]. Abnormal DNA methylation is not only a late-stage feature of tumors, but also a drives the early development of tumors [14]. Aging, diet, obesity, *H. pylori* and Epstein Barr virus infection are all associated with gene methylation in GC [15]. Studies confirmed the involvement of DNA methylation in the transformation of the normal gastric mucosa to GC [16]. The tumor-related genes are hypermethylated in precancerous lesions and intestinal metaplasia [17, 18], and inhibiting DNA methylation reduces the incidence of GC in animal models [19, 20], DNA methylation could be used as a biomarker for diagnosis, treatment and prognosis of GC [15, 21]. DNA methylation research has opened a new field in genomics biology by identifying novel biomarkers for several cancers, such as gastrointestinal tumors, breast and lung cancer, malignant melanoma, and other tumors [22–27]. In one study, hypermethylation of the potassium calcium-activated channel subfamily M alpha 1 (KCNMA1) promoter was detected in 68.7% of GC tissues examined and related to shorter survival time. KCNMA1 hypermethylation was implicated as an independent prognosis factor in GC [28]. By analyzing the genome-wide methylation status, Simon et al. [29] showed that a multiple-DNA methylation signature could predict the therapeutic outcome of bevacizumab during metastatic breast cancer treatment, and was the first DNA methylation study that precited the efficacy of bevacizumab as a cancer treatment. Emerging evidence indicates that DNA methylation is closely correlated with the development, invasion, and metastasis of cancer, and could hence, be a valuable prognostic biomarker [14, 24].

We analyzed the genome-wide methylation map of GC tissues in The Cancer Genome Atlas (TCGA) database to explore the use of DNA methylation as a prognostic biomarker. The Kaplan–Meier survival curve and receiver operating characteristic (ROC) analyses were used to determine the specificity and sensitivity of a four-DNA methylation signature as a prognostic biomarker. The independence and repeatability of this signature as a prognostic factor were verified in validation datasets.

## Methods

### GC DNA methylation data from the TCGA dataset

Level three DNA methylation data of patients with GC obtained using the Infinium HumanMethylation450 BeadChip (Illumina Inc., CA, USA) were retrieved from the TCGA database. The CpG sites in the genome were defined following the guideline of the Genome Reference Consortium Human Build 38 (GRCh38). The DNA methylation expression were assessed by β-values and calculated as the ratio of M and M + U, where M represents the signal from methylated beads targeting CpG site, while U represents the signal from unmethylated beads. Data for a total of 368 GC patients comprising 485,578 DNA methylation sites were included after excluding data for patients with a short survival time (< 30 days) and cases for whom clinical survival information was lacking. The relationship between DNA methylation levels and the corresponding survival time of patients with GC was analyzed. The 368 patients with GC were divided evenly into the training (n = 184) and validation set (n = 184) to cross-validate the prognostic indicators. The training set was used to construct the prognostic model, and the validation set was used to evaluate the prognostic accuracy of the model for the survival status of patients with GC. There were no significant differences in the sets concerning clinicopathological data.

### Statistical analyses

Univariate Cox regression analysis was conducted in the training set based on the OS time and the CpG expression in patients with GC. A total of 1274 methylation sites that were significantly related to GC prognosis (P < 0.005) were screened for the prognostic methylation markers. To avoid overfitting of the prognostic model, LASSO regression analysis (Glmnet R package) and cross-validation were first performed; the 1274 methylation sites were screened more than 1000 times. From the screened methylation sites, if specific sited were detected more than 800 times, they were regarded as candidate biomarkers and were analyzed further by multivariate Cox regression analysis designed to identify the independent prognostic biomarkers by controlling confounding factors or covariates. Risk score models were constructed based on the risk coefficient and expression of methylation sites. The median risk score was regarded as the cutoff point. Patients with GC were categorized into “high-risk” or “low-risk” groups according to a high and low score, respectively. Log-rank testing of the Kaplan–Meier curve was performed to calculate the difference in OS of the two groups. We conducted ROC analysis for patients with OS < 3 years in terms of the methylation biomarkers. The area under the ROC curve (AUC) was calculated with 95% confidence intervals (CIs) to assess the predictive accuracy of the biomarkers. The Z-test further compared the AUCs of these diverse biomarkers. All statistical analyses were performed using R software (version 3.4.4).

## Results

### Clinical characteristics of patients

The 368 patients with GC from the STAD cohort of the TCGA database who had been diagnosed clinically and pathologically were analyzed. The median age was 67 years (range, 58–73 years) and 66.6% of the patients were male (Table 1). The median OS was 489 days (range, 31–3720 days) encompassing the different pathological stages of GC. The 3-year OS rate of all patients was 13.3%. Based on the pathological characteristics of TNM staging of GC, histological grade, histological type, and anatomical subdivision following the World Health Organization criteria, the majority of the STAD cohort of GC were diagnosed initially with invasive disease (T3–4, 74.7%), lymphatic metastasis (69.3%), and extensive malignancy (G3–4, 62.2%). The occurrence rate of the intestinal type and unspecific type of GC was 46.5% and 32.3%, respectively, and were higher than the rate of the diffuse type. Anatomic subdivisions of GC were obtained from different regions, such as gastroesophageal junction (GEJ), cardia fundus, and antrum. Among them, stomach antrum (36.4%) and fundus (36.4%) were the predominant sites for GC (Table 1).

**Table 1:** Clinicopathological characteristics of participants in TCGA cohort

Characteristics	Groups	Patients		
		Total (N = 368)	Training dataset (N = 184)	Validation dataset (N = 184)
		Sample (n, %)	Sample (n, %)	Sample (n,%)
Age at diagnosis	Median (IQR)	67.00 (58.00, 73.00)	67.50 (58.00, 73.00)	66.00 (58.00, 73.00)
Sex	Female	123 (33.4)	62 (33.7)	61 (33.2)
	Male	245 (66.6)	122 (66.3)	123 (66.8)
Live status	Living	226 (61.4)	113 (61.4)	113 (61.4)
	Death	142 (38.6)	71 (38.6)	71 (38.6)
Pathologic T	T1–2	93 (25.3)	44 (23.9)	49 (26.6)
	T3–4	275 (74.7)	140 (76.1)	135 (73.4)
Pathologic N	N0	113 (30.7)	58 (31.5)	55 (29.9)
	N1–3	255 (69.3)	126 (68.5)	129 (70.1)
Pathologic M	M0	334 (90.8)	165 (89.7)	169 (91.8)
	M1–3	34 (9.2)	19 (10.3)	15 (8.2)
Pathological stage	I–II	166 (45.1)	86 (46.7)	80 (43.5)
	III–IV	202 (54.9)	98 (53.3)	104 (56.5)
Histologic grade	G1–2	139 (37.8)	70 (38.0)	69 (37.5)
	G3–4	229 (62.2)	114 (62.0)	115 (62.5)
Histological type	Diffuse	78 (21.2)	39 (21.2)	39 (21.2)
	Intestinal	171 (46.5)	86 (46.7)	85 (46.2)
	NOS	119 (32.3)	59 (32.1)	60 (32.6)
Anatomic subdivision	Antrum	134 (36.4)	66 (35.9)	68 (37.0)
	Cardia	49 (13.3)	27 (14.7)	22 (12.0)
	Fundus	134 (36.4)	66 (35.9)	68 (37.0)
	GEJ	42 (11.4)	20 (10.9)	22 (12.0)
	NOS	9 (2.4)	5 (2.7)	4 (2.2)

### Methylation markers associated with prognosis of GC patients in training set

**Fig. 1:**
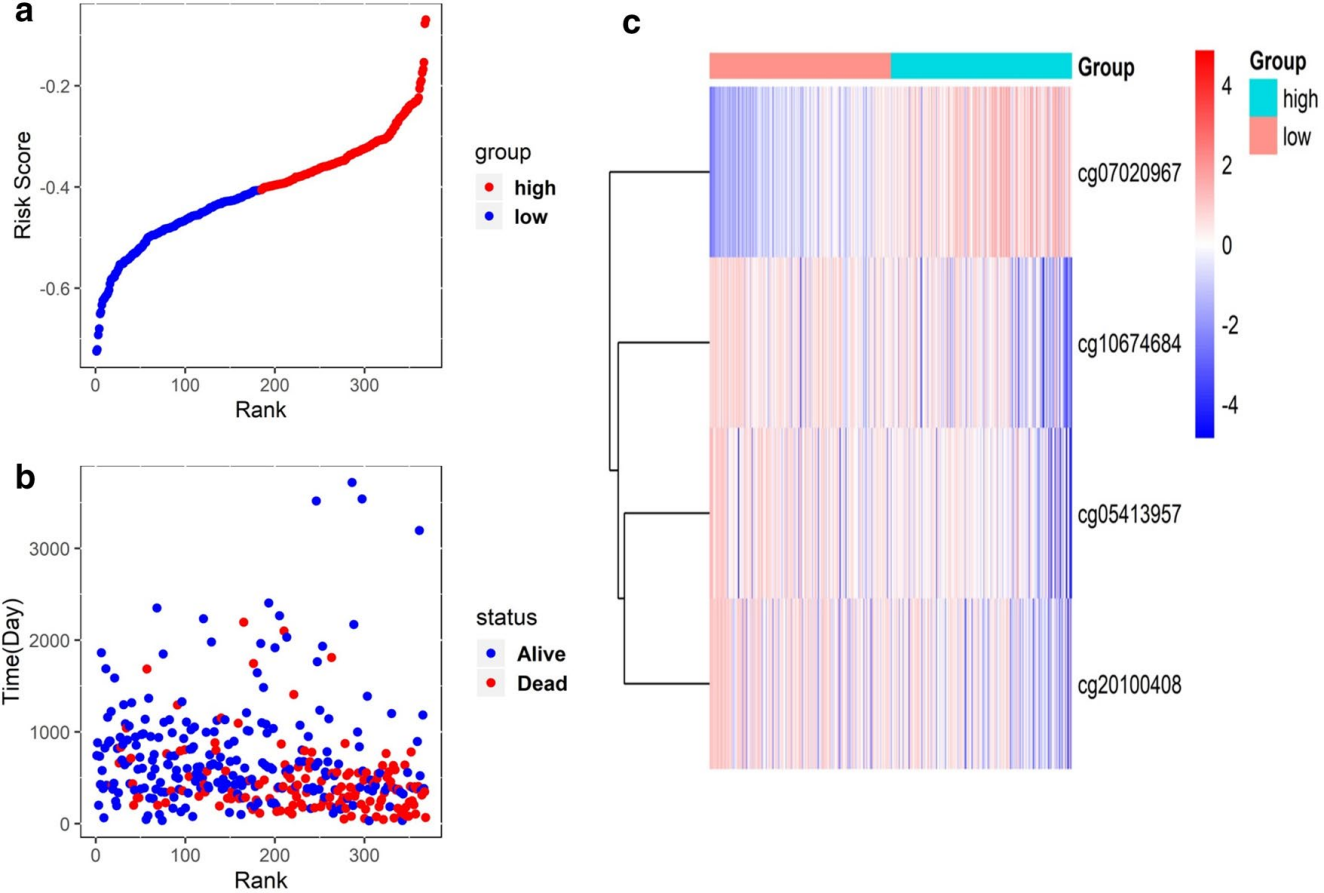
Development of the prognostic index based on prognosis‐related DNA methylation sites. **a** Rank of prognostic index and distribution of groups. **b** Survival status of patients in different groups. **c** Heatmap of expression profiles of included DNA methylation sites

From the DNA methylation expression data in patients with GC, the DNA methylation markers related to the OS of patients with GC in the training set were screened by univariate Cox proportional hazard regression analysis (Fig. 1a–c). A total of 1274 DNA methylation sites correlated significantly with the prognosis of patients with GC (P < 0.005). Of these, four methylation sites including cg05413957 (GRID2IP), cg07020967 (TMEM117), cg10674684 (intergenic region), and cg20100408 (HLA-DP) were selected as the optimal model sites for the prognostic assessment of patient with GC by LASSO regression and multivariate Cox regression analysis. The risk scoring formula was calculated as follows: Risk score = − 0.33126 × β value of cg05413957 + 0.53604 × β value of cg07020967 − 0.38673 × β value of cg10674684 − 0.21447 × β value of cg20100408. The scores revealed strong associations of poor prognosis with hypermethylation of cg07020967 and the hypomethylation of cg05413957, cg10674684, and cg20100408 sites.

### Association between the four-DNA methylation biomarker and prognosis

**Fig. 2:**
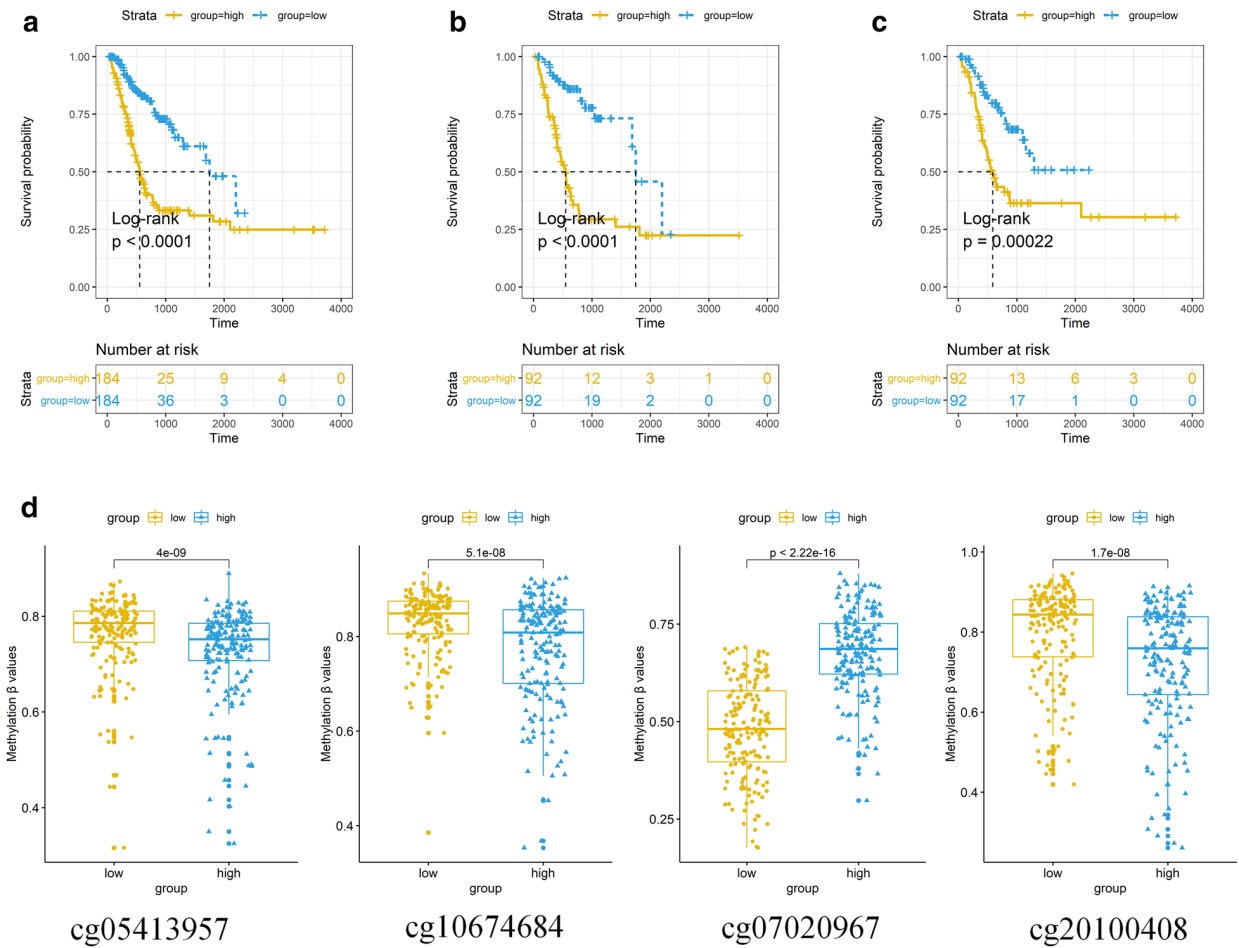
The overall survival (OS) and methylation levels of gastric cancer patients, The Kaplan–Meier curve of the OS for high-risk and low-risk scores ranking by the four-DNA methylation indicator in the training dataset (N = 184) (**a**), the validation dataset (N = 184) (**b**) and all cohort dataset (**c**). The log-rank test illustrated the higher risk scores are significantly associated with worse OS (P < 0.001). **d** The differential expression level of methylation sites in training dataset of GC cohort. Mann–Whitney U test was used to evaluate the differences between the high-risk score and low-risk score

**Fig. 3:**
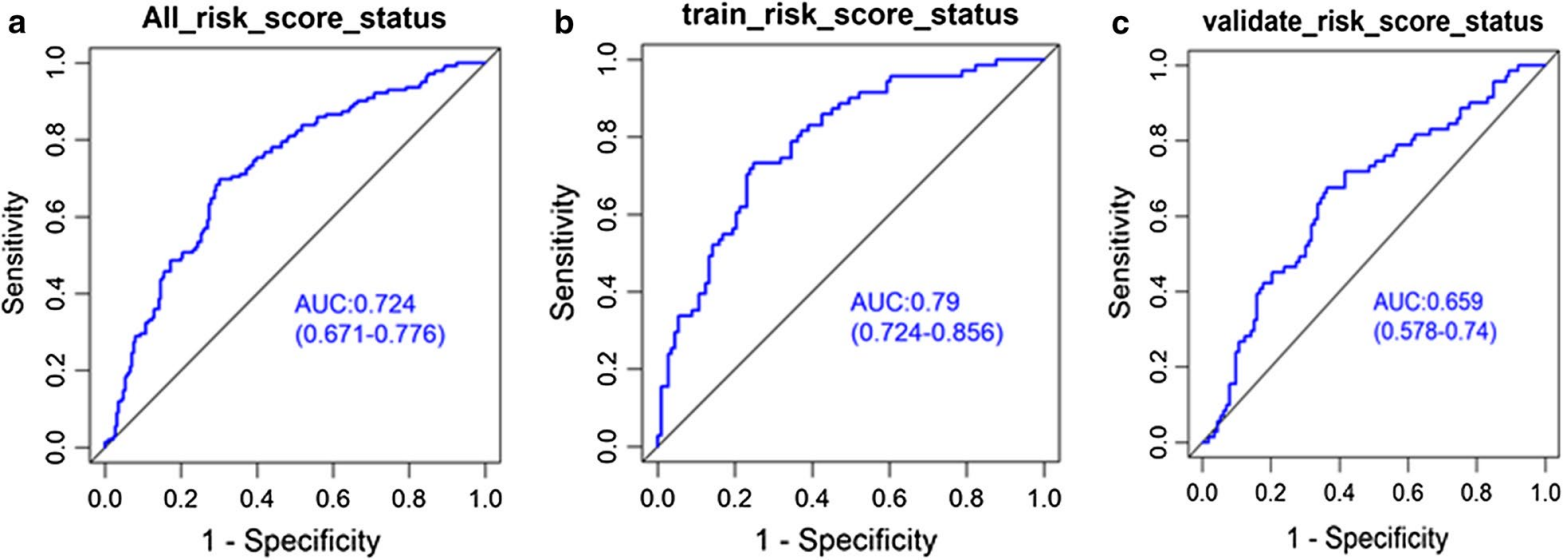
ROC analysis of the four-DNA methylation biomarker for predicting the OS of GC patients in total dataset (**a**), the training dataset (**b**) and the validation dataset (**c**)

Hazard ratios (HRs) obtained from the Cox regression analysis showed a relation between the four-DNA methylation signature and the OS (P < 0.0001, HR 2.72, 95% CI 2.127–3.474). The prognostic value of the four-DNA methylation signature was explored by Kaplan–Meier survival analyses of the training and validation datasets. The median risk scores as cutoff value were selected to define the high-risk and low-risk for GC prognosis. As shown in Fig. 2a–c, survival was significantly longer in the low-risk group compared to the high-risk group in the training dataset (P < 0.001) and the validation dataset (P < 0.001). A similar result was found in the all-cohort dataset (P < 0.001). The findings indicated the utility of the four biomarkers as prognostic indicators in patients with GC. The differential expressions of the four methylation biomarkers were analyzed individually, and distinct, aberrant regulation was observed (Fig. 2d). Three methylation sites (cg05413957, cg10674684, and cg20100408) were obviously downregulated in the high-risk group compared to the low-risk group, whereas the expression of cg07020967 was higher in the high-risk group compared to the low-risk group in the training and validation datasets. The predictive performance of the four-DNA methylation signature was assessed ROC analysis. The AUC of the four-DNA methylation signature was 0.72 (95% CI 0.67–0.78) in the total cohort, 0.79 (95% CI 0.72–0.86) in the training dataset and 0.66 (95% CI 0.58–0.74) in the validation dataset (Fig. 3a–c), implying the high predictive accuracy of four-DNA methylation signature for GC prognosis.

### Predictive performance of the four-DNA methylation biomarker in different patient subgroups

Many confounding clinicopathological factors could influence the predictive performance of the four-DNA methylation biomarker. Subgroup analyses were conducted on the TNM stage, histologic type, histologic grade, and anatomic subdivisions to gauge the clinical applicability and predictive accuracy of the four-DNA methylation biomarker for prognosis of GC with regard to different clinicopathological characteristics (Table 2). The biomarker in the high-risk group of males and females associated significantly with poor prognosis, compared to the case for the low-risk group (both P < 0.001). The AUC value of prognostic accuracy for sex was 0.69 and 0.68, respectively (Additional file 1: Figure S1). The risk scoring system could be applied to the extent of infiltration, with discrimination of extensive infiltration (T3–4) from scant infiltration (T1–2), to lymphatic infiltration, with discrimination of lymphatic metastasis (N1–3) from non-lymphatic metastasis (N0), with an AUC value of 0.67 and 0.72, respectively. Risk score was also valuable in discriminating non-distant organ metastasis (M0) from extensive metastasis (M1–3). However, the risk score could not discriminate the survival time of M1–3 Patients with GC (Additional file 1: Figure S2). Although the risk scoring system could be applied to the entire pathologic stage and histologic grade for OS prediction, the predictive accuracy was lower than that for the pathologic stage III-IV (AUC: 0.60 vs. 0.72) and histologic grade G3–4 (AUC: 0.64 vs. 0.70) (Additional file 1: Figure S3–4). Concerning anatomic subdivisions, in addition to the non-specific classification, the risk scoring system was valuable for prediction of OS for cancers from the antrum, fundus, cardia, and GEJ of the stomach. Survival was worse for high-risk score patients than for the low-risk score patients in the antrum group (P = 0.003), cardia group (P = 0.005), fundus group (P = 3.79E−08), and GEJ group (P < 0.001). The respective AUC values of 0.59, 0.71, 0.75, and 0.82 indicated the predictive accuracy of the four-DNA methylation signature (Additional file 1: Figure S5). The collective results support the application of the signature as an independent prognostic predictor of survival in patients with GC.

**Table 2:** Results of Kaplan–Meier and ROC analysis based on different subgroups

Characteristic	Group	Sample size	Kaplan–Meier P value	AUC	CI 95%
Sex	Female	123	P < 0.001	0.69	0.37–0.78
	Male	245	3.65E−08	0.68	0.49–0.84
Pathologic T	T1–2	93	0.04	0.67	0.44–0.85
	T3–4	275	1.77E−11	0.67	0.48–0.85
Pathologic N	N0	113	0.17	0.53	0.36–0.76
	N1–3	255	7.72E−10	0.72	0.44–0.81
Pathologic M	M0	334	4.36E−10	0.68	0.51–0.77
	M1–3	34	0.09	0.64	NA–NA
Pathological stage	I–II	166	0.001	0.60	0.42–0.79
	III–IV	202	6.91E−07	0.72	0.48–0.83
Histologic grade	G1–2	139	0.002	0.64	0.57–0.88
	G3–4	229	9.17E−10	0.70	0.38–0.74
Anatomic subdivision	Antrum	134	0.003	0.59	0.30–0.81
	Cardia	49	0.005	0.71	0.085–1.0
	Fundus	134	3.79E−08	0.75	0.50–0.95
	GEJ	42	P < 0.001	0.82	NA–NA
	NOS	9	0.35	0.92	NA–NA

### Comparison of the four-DNA methylation signature with other known prognostic biomarkers

**Fig. 4:**
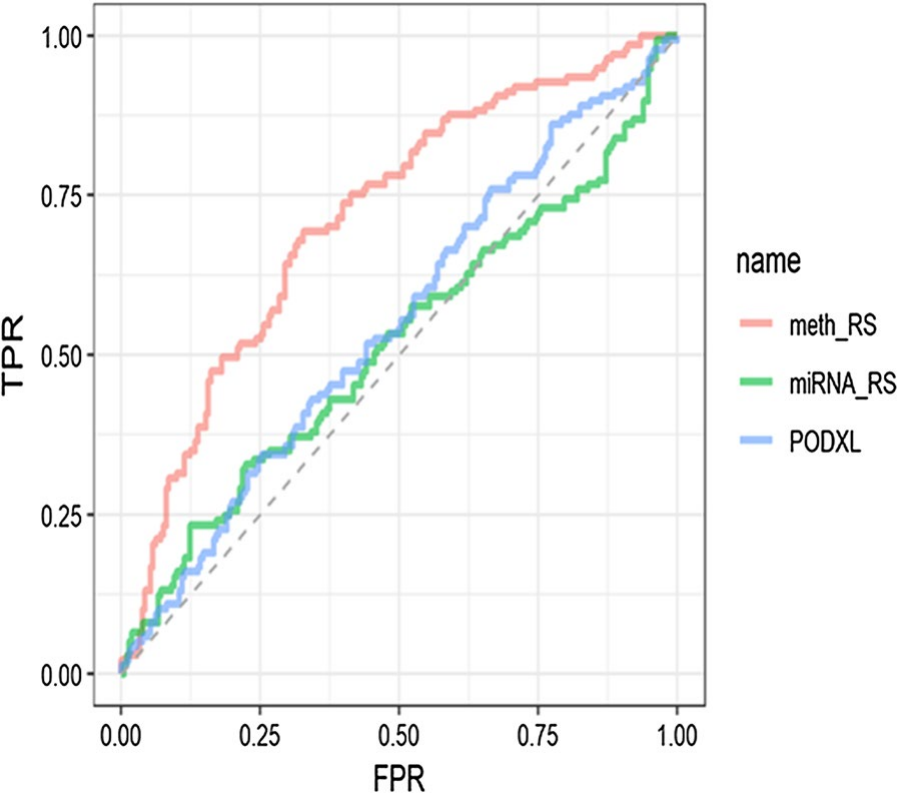
ROC curves show the AUC of the four-DNA methylation biomarker (0.71, 95% CI 0.66–0.77) and other known biomarker such as miRNAs (0.52, 95% CI 0.45–0.58) and PODXL (0.55, 95% CI 0.49–0.61) in predicting the OS of GC patients, meth_RS: methylation ROC analysis, miRNA_RS: miRNA ROC analysis

Prior studies identified some independent prognostic biomarkers for GC. For example, an analysis of microRNA (miRNA) expression profile and risk score identified a seven-miRNA biomarker (miR-10b, miR-223, miR-30a-5p, miR-126 miR-21, miR-338, and let-7a) for OS prediction, and was validated using an independent dataset [30]. An examination of the Oncomine GC database and 32 paired fresh GC tissues revealed upregulated expression of podocalyxin-like protein 1 (PODXL). PODXL downregulation could inhibit tumor development, as shown by the inhibition of epithelial mesenchymal transformation, invasion, and metastasis in vitro and reduction of tumorigenesis in vivo. The Cox proportional hazards model showed PODXL as an independent prognostic biomarker for OS of patients with GC [31]. To verify the predictive performance of the four-DNA methylation indicator, we evaluated the sensitivity and specificity of the seven-miRNA signature and PODXL from other studies in our dataset. Comparison of AUCs revealed that the four-DNA methylation indicator was superior to other known prognostic biomarkers, including mRNA, miRNA, multi-/single-molecule models (Fig. 4). A statistical comparison using the Z-test indicated that the four-DNA methylation indicator had significantly higher (P < 0.05) predictive performance compared to other known biomarkers (Additional file 1: Table 1S). Concerning the prediction of the OS of patients with GC, all the results evidenced that the four-DNA methylation indicator showed a better predictive ability.

## Discussion

GC is a highly heterogeneous disease involving a complex interplay between host and environmental risk factors including *Helicobacter pylori*, alcohol consumption, tobacco smoking, dietary habits, and others [1]. GC tumorigenesis and progression involve complex regulatory networks [4]. Consequently, multi-molecule models are superior to single-molecule models for the diagnosis and prognosis of GC. DNA methylation is a reliable molecular biomarker that can easily be evaluated by PCR. It is an appropriate biomarker for non-invasive clinical measurements of blood and other body fluids and feces [21, 26, 32, 33]. For instance, methylation of CDH1 could be detected in tissue samples, preoperative peritoneal washes, and serum of patients with GC [32–34]. The aberrant methylation of Vimentin in feces is the basis of a commercial test for colorectal cancer [35]. Here, we analyzed the whole genome-wide methylation map in patients with GC. DNA methylation sites that correlated with OS in GC patients were screened, and the candidate biomarkers were used to construct a model for predicting survival time in patients with GC using the high-risk and low-risk scores. Cross-validation of the training and validation datasets tested the stability of the model for prediction of GC prognosis. ROC analysis showed that the four-DNA methylation signature had a superior predictive ability compared to the other tested biomarkers.

Currently, several biomarkers for GC prognosis are available and include mRNAs, non-coding RNAs, and proteins [36–39]. Long Intergenic Non-Protein Coding RNA 1133 (LINC01133) was identified as a potential prognostic biomarker for patients with GC. LINC01133 inhibits GC progression and metastasis by sponging miR-106a-3p to regulate APC expression and the Wnt/β-catenin pathway [38]. Many studies have established Her-2 as a prognostic biomarker for GC, which informed the development of targeted drugs, such as trastuzumab to treat Her-2 positive GC [40, 41]. Negatively regulated by KLF4, PODXL is a novel prognostic biomarker of GC [31]. The seven-miRNA signature was shown to be an independent biomarker of relapse-free survival and OS in GC [30]. However, some studies lacked large-scale cohort validation, and it was unclear whether the miRNA signature was an independent prognostic indicator with high predictive ability. In this study, using the methylation data from the STAD database, the four-DNA methylation sites related to OS of patients with GC were analyzed via the construction of a regression model. The predictive accuracy of this biomarker was assessed in training and validation datasets by ROC analysis. The interaction of variables was controlled by subgroup analyses, including sex, TNM stages, histologic grade, and anatomic subdivision. The four-DNA methylation biomarker could differentiate early- and advanced-GC and was a novel and independent biomarker with good predictive ability. Additionally, comparisons with the seven-miRNA signature and PODXL demonstrated the superior efficiency of the four-DNA methylation biomarker with regard to the predictive performance.

Three candidate sites of the four-DNA methylation signature were from corresponding genes of GRID2IP (cg05413957), TMEM117 (cg07020967) and HLA-DP (cg20100408). However, the methylation site of cg10674684 did not match to the definite gene due to the intergenic region. GRID2IP is expressed predominantly at the post-synapse regions of parallel fiber-Purkinje cells in the brain and is crucial for synaptogenesis and synaptic plasticity. The formation of excitatory synapses between neurons and tumor cells promotes cancer growth. The molecular chemical signals released from these pseudo-synapses are a likely cause of breast cancer brain metastasis [42–45]. An analysis of the TCGA database has also implicated GRID2IP as a novel prognostic marker in patients with cholangiocarcinoma [46]. TMEM117 is a six transmembrane protein with a conserved sequence; it is present in pro- and anti-apoptotic BCL-2 family of proteins. Bioinformatic analysis revealed an association between TMEM117 downregulation and pancreatic cancer tumorigenesis and metastasis [47], trans-differentiation to mesenchymal cells in breast cancer [48], phenotypic change of normal cells of gliomas [49], and development of malignant lymphoblastic leukemia [50]. However, experiment data are insufficient to support a role of TMEM117 in cancers, further research is required to identify the tumorigenic role of TMEM117 protein levels in cancer. The HLA-DP protein/peptide-antigen receptor and graft-versus-host disease antigen is an HLA class II beta chain paralog. It is a heterodimer comprised of an alpha chain (DPA) and beta chain (DPB) that are both anchored to the membrane as HLA-DPB1. The biological involvement of HLA-DPB is mainly in the immune response. HLA-DP is participates in the origin and development of cervical cancer, and different HLA-DP polymorphisms correspond to different cervical cancer risks in the Chinese and Swedish populations [51]. Genetic polymorphism of HLA-DP is also associated with the prognosis of Hodgkin’s lymphoma [52]. Although the three genes (GRID2IP, TMEM117, HLA-DP) are involved in the progression of cancers, their role in GC is unclear. Because of the limitations about bioinformatics tools when analyzed large amounts of data, and disadvantages of Infinium HumanMethylation450 BeadChip (Illumina Inc., CA, USA) [53–55], we needed validate bioinformatics result by experiment. In the future, We will verify the expression level and predictive efficacy of the four methylation sites in GC cells, mouse models and tissues to explore the genetic mechanism underlying the development of GC.

## Conclusion

We established a GC prognostic classifier model consisting of four CpG sites and validated the model in training and validation datasets. The four-DNA methylation signature serves as an biomarker to evaluate the prognosis of patients with GC. The signature remains to be validated in a large-scale cohort and through basic research studies (Additional file 1: Supplemental Figure S1–5 and Supplemental Table S1).

## Supplementary information

**Additional file 1. Supplemental Figure S1–S5 and Supplemental Table S1.**

## Data Availability

Additional data not presented in the manuscript can be obtained by contacting the authors.

## Abbreviations

GC: Gastric cancer
TCGA: The Cancer Genome Atlas
ROC: Receiver operating curve
OS: Overall survival
CI: Confidence interval
AUC: Area under the curve
GEJ: Gastroesophageal junction
HR: Hazard ratios
LASSO: The least absolute shrinkage and selection operator
TNM: Tumor node metastasis
STAD: Stomach adenocarcinoma
