# Supplementary material for: A four-DNA methylation signature as a novel prognostic biomarker for survival of patients with gastric cancer
Source: Cancer Cell Int. 2020 Mar 20;20:88. doi: 10.1186/s12935-020-1156-8 (PMC7085204; doi:10.1186/s12935-020-1156-8)
Supplement: Supplementary file 1 — Additional file 1. Supplemental Figure S1–S5 and Supplemental Table S1. [file 12935_2020_1156_MOESM1_ESM.doc]

**A four-DNA methylation signature as a novel prognostic biomarker predicts the survival of gastric cancer patients**

Chunmei Li^1,2#^, Ya Zheng^1,3#^, Ke Pu^1,3#^, Da Zhao^2^, Yuping Wang^1,3^, Quanlin Guan^4^, Yongning Zhou^1,3*^

**Figure S1**. Kaplan-Meier and ROC analyses of GC patients in different cohorts regrouping by patient sex (female and male). **a.** Kaplan-Meier analysis with two-sided log-rank test was performed to assess the differences in OS between the low-risk and high-risk patients. **b.** ROC curves were to evaluate the predicting accuracy of four-DNA methylation biomarker for the prognosis of GC patients.


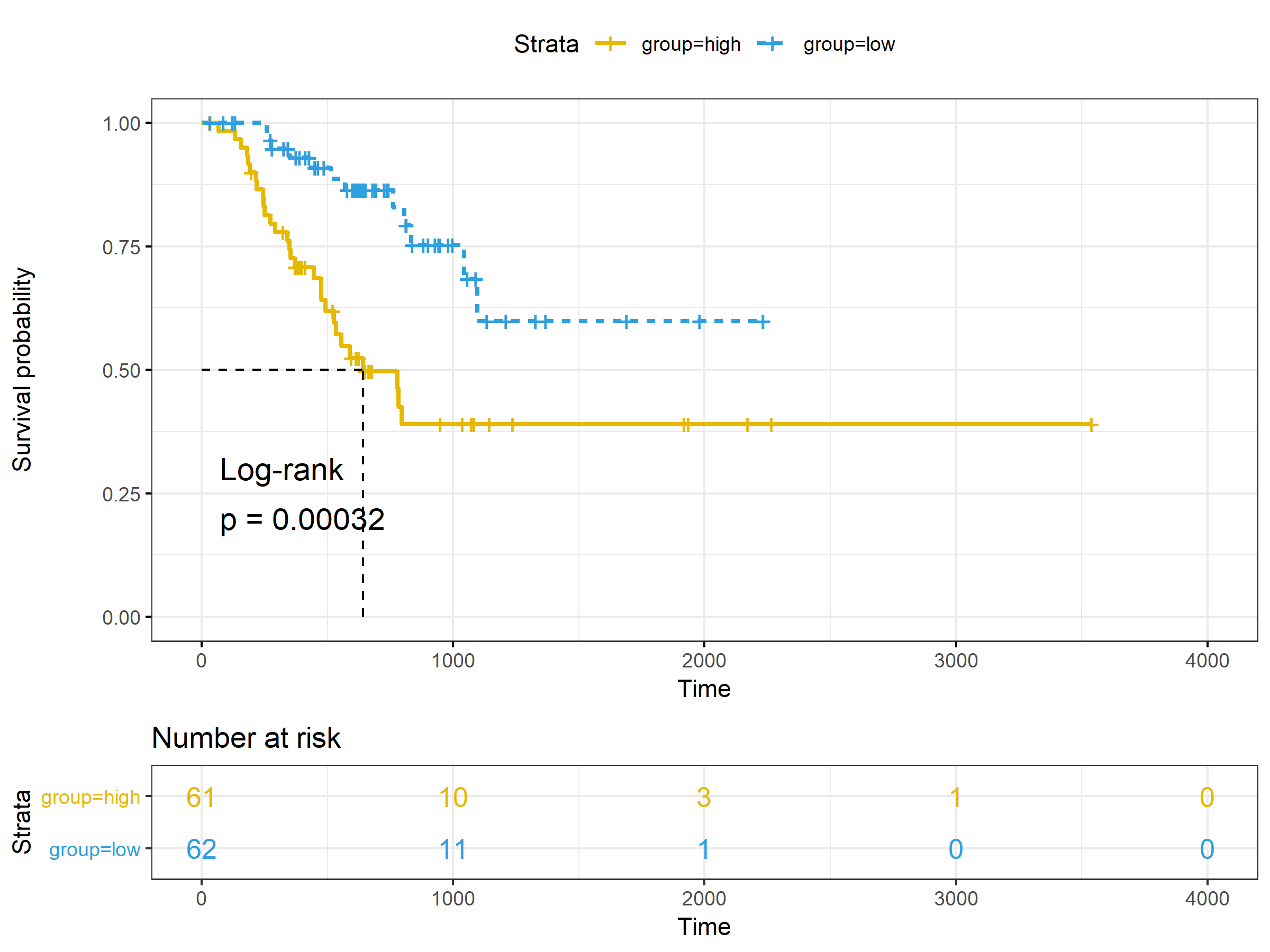

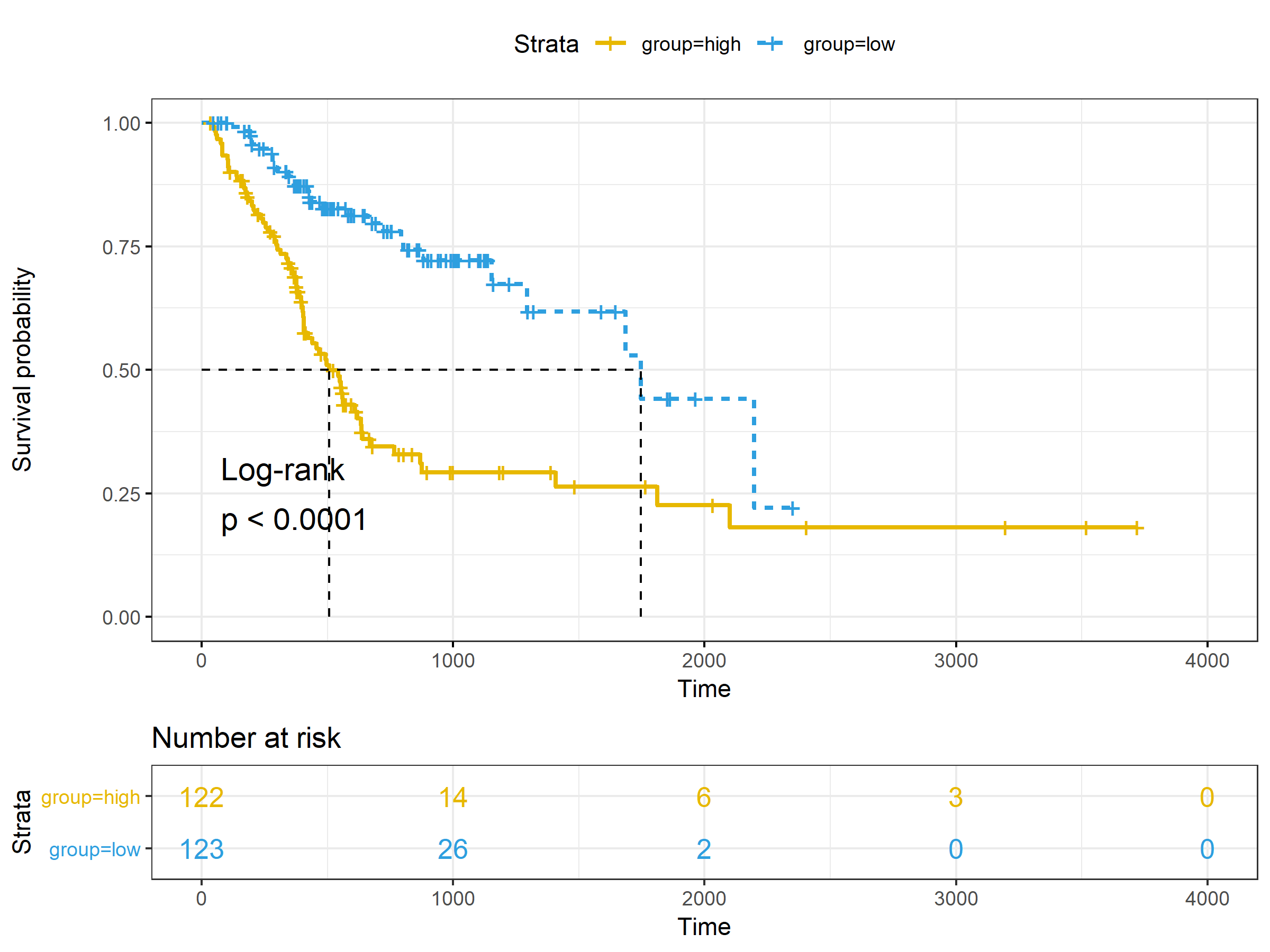


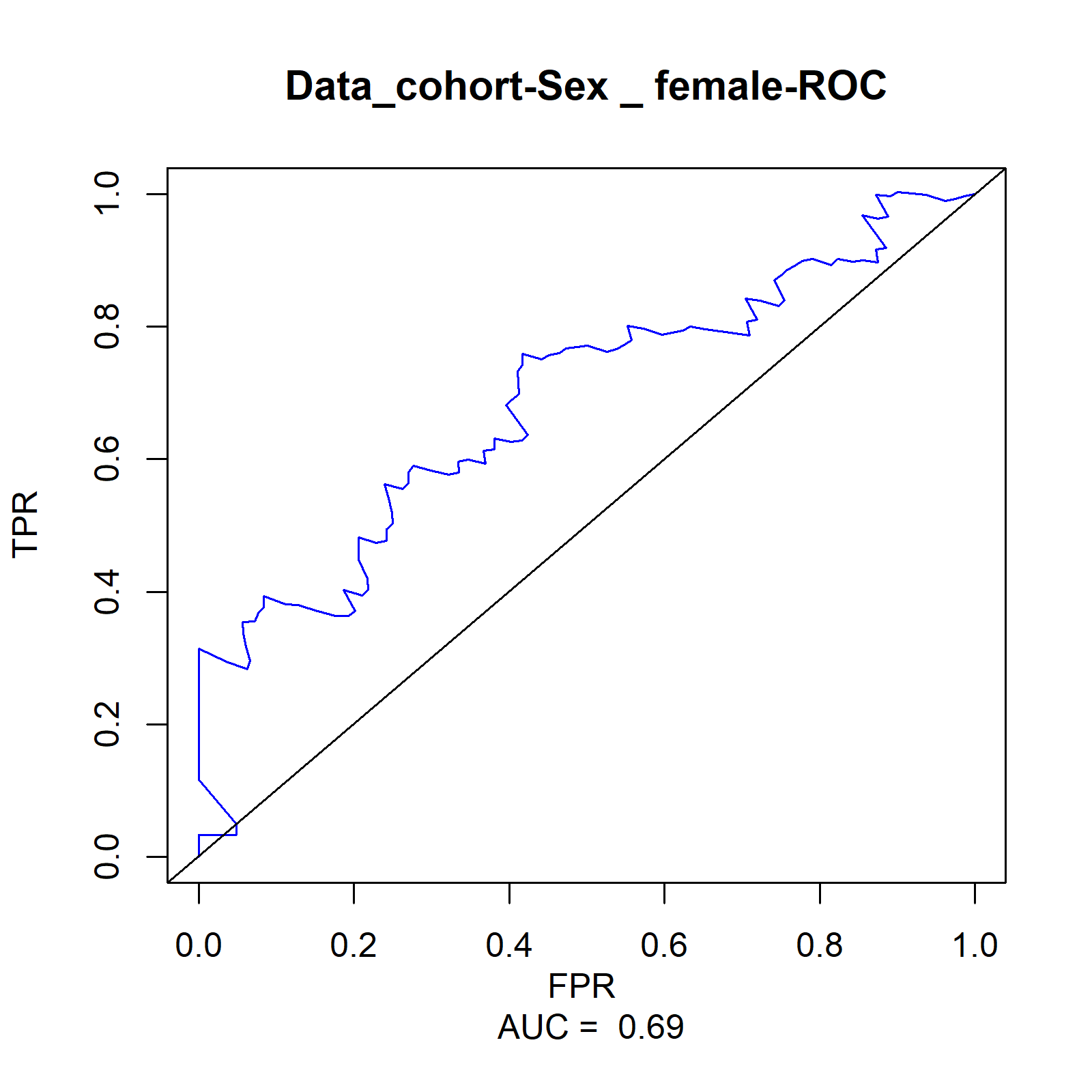

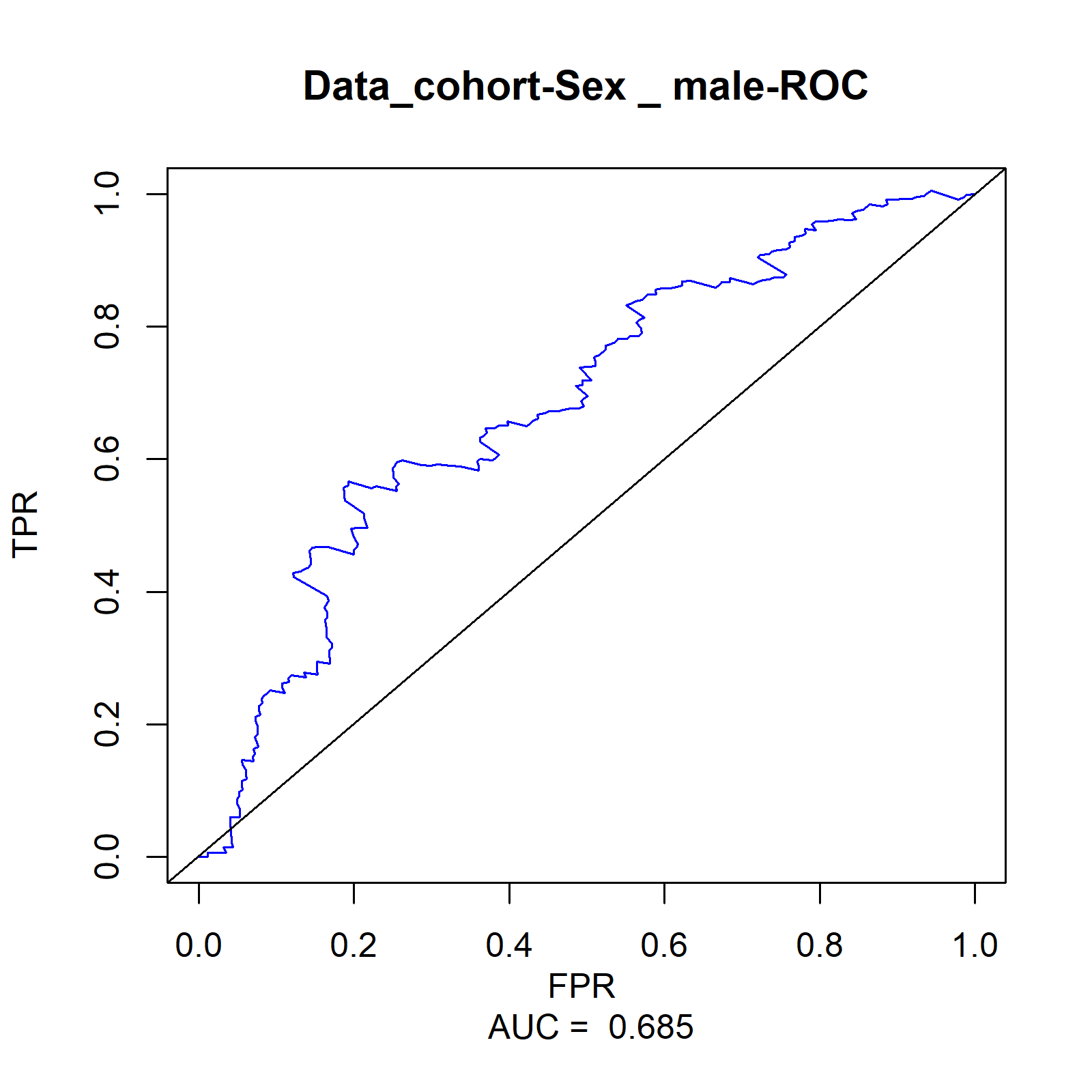


Female and male

**Figure S2**. Kaplan-Meier and ROC analyses of GC patients in different cohorts regrouping by TNM stage of GC (T_1-2_/T_3-4_, N_0_/N_1-3_ and M_0_/M_1-3_). **a.** Kaplan-Meier analysis with two-sided log-rank test was performed to assess the differences in OS between the low-risk and high-risk patients. **b.** ROC curves were to evaluate the predicting accuracy of four-DNA methylation biomarker for the prognosis of GC patients.


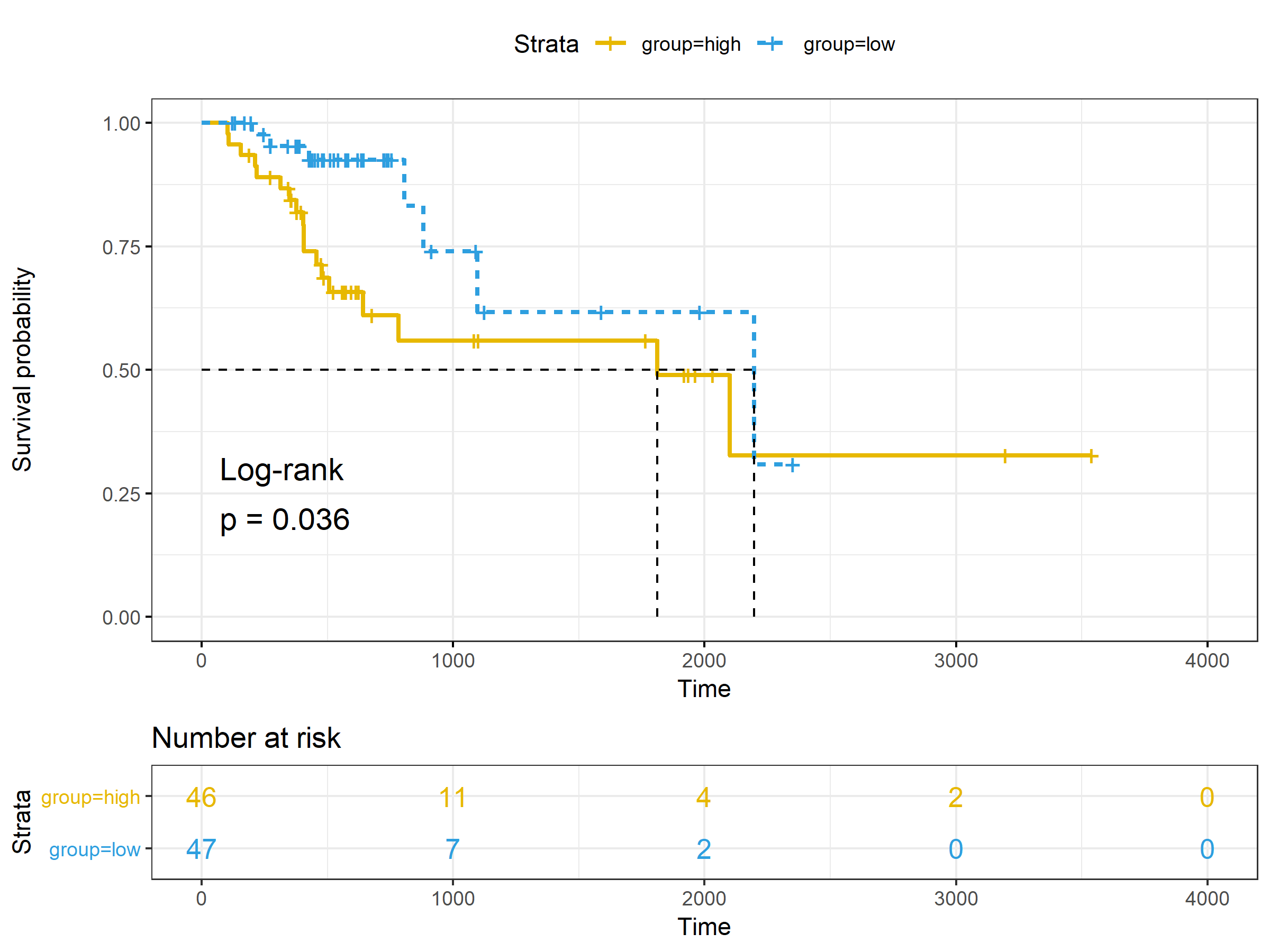

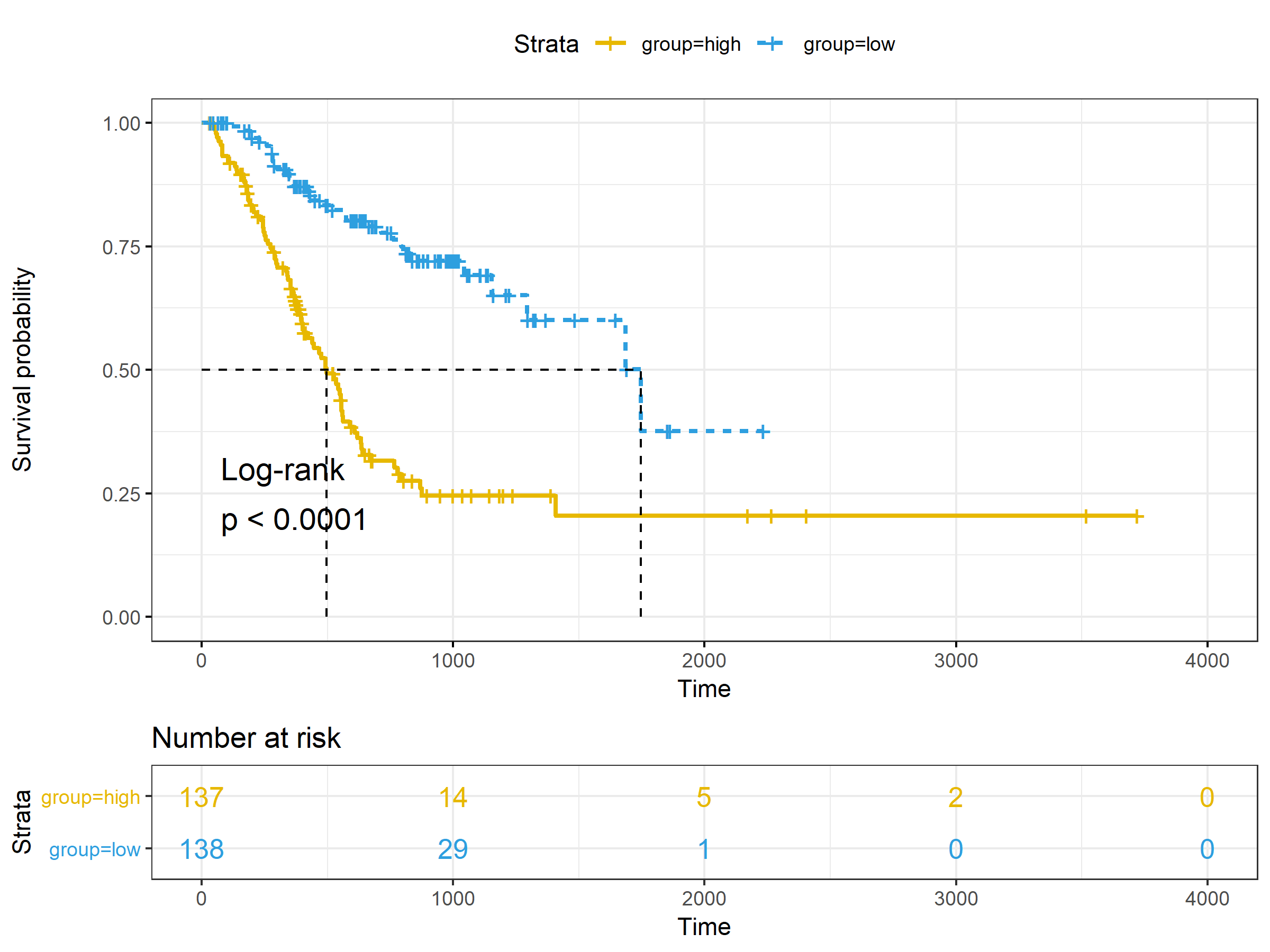


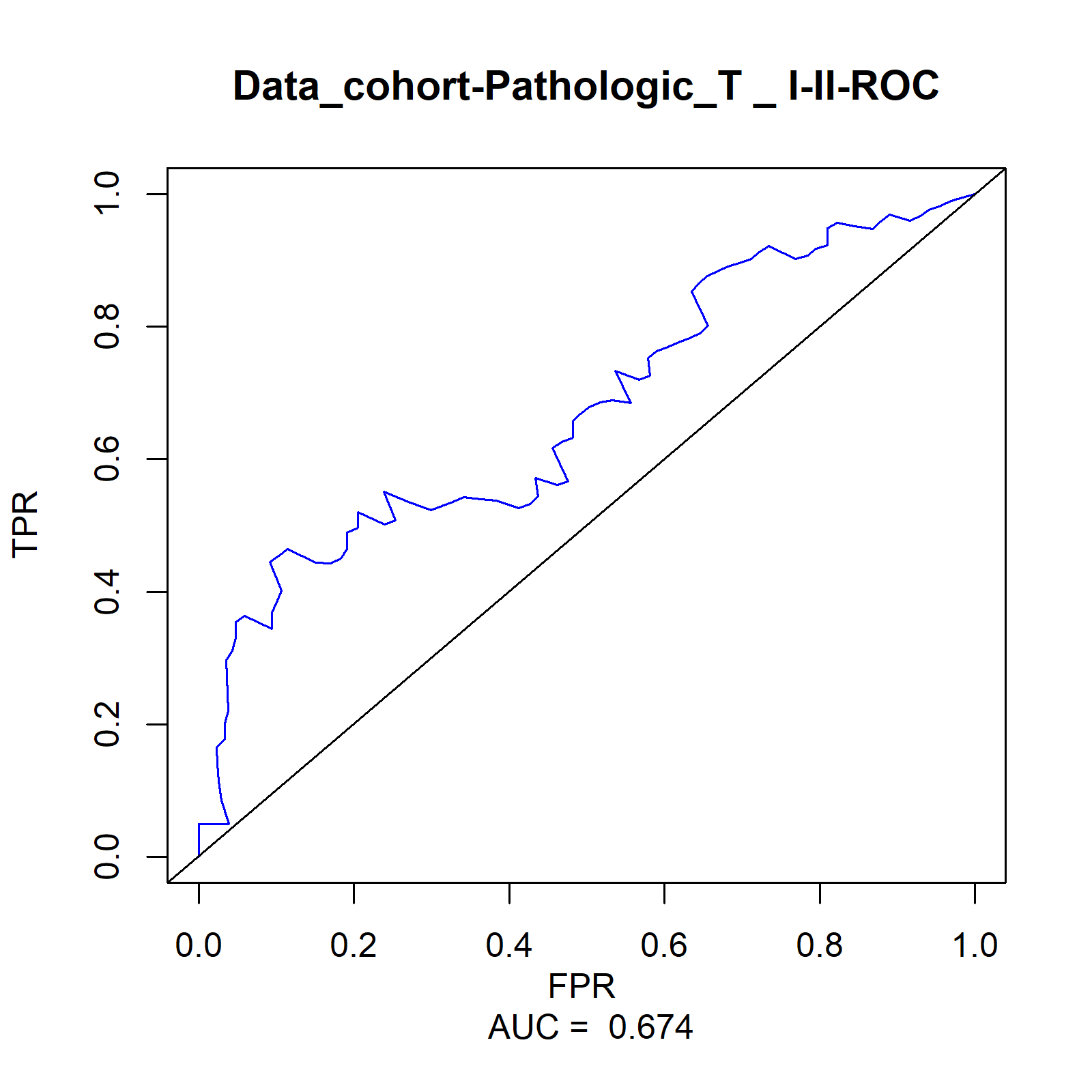

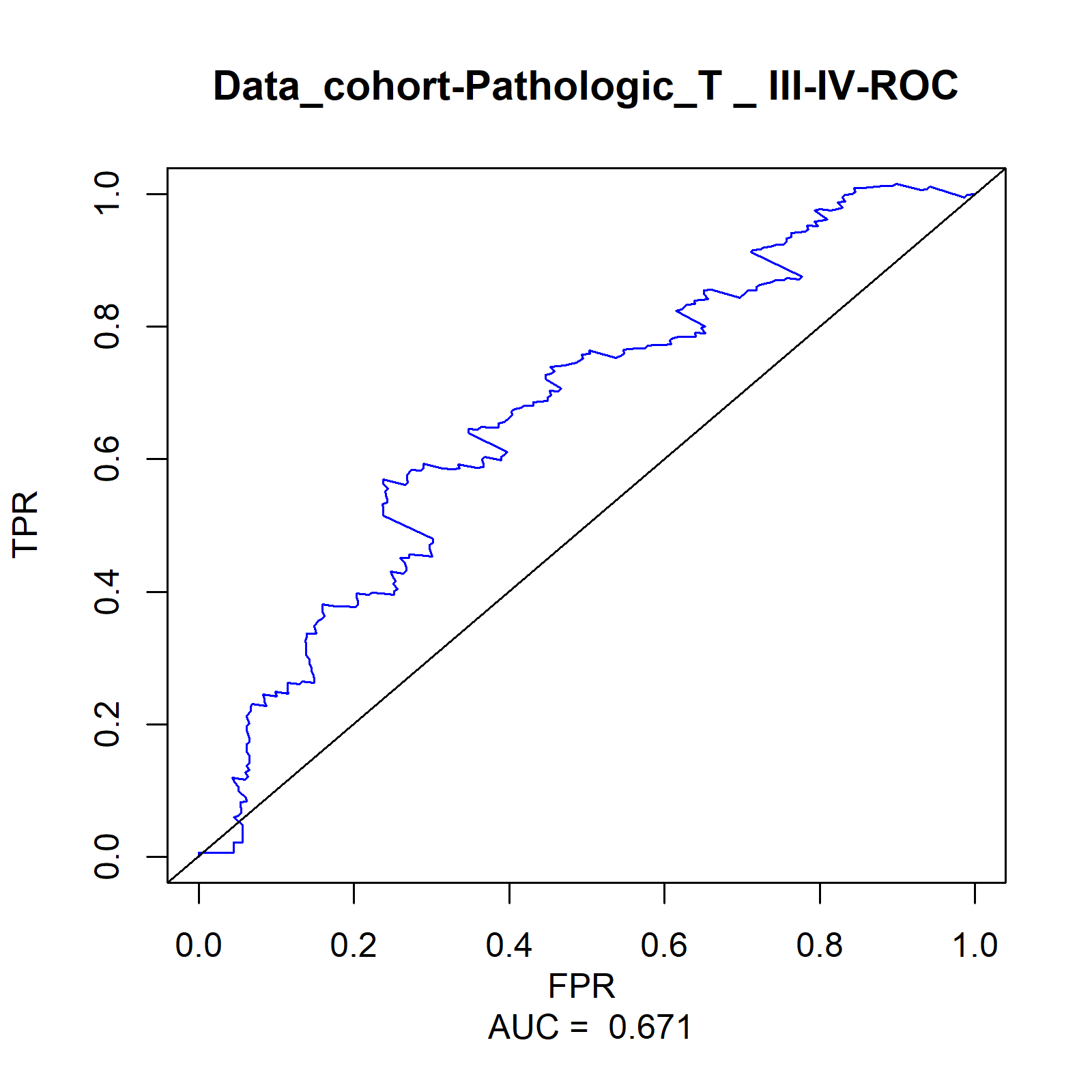


T_1-2_/T_3-4_


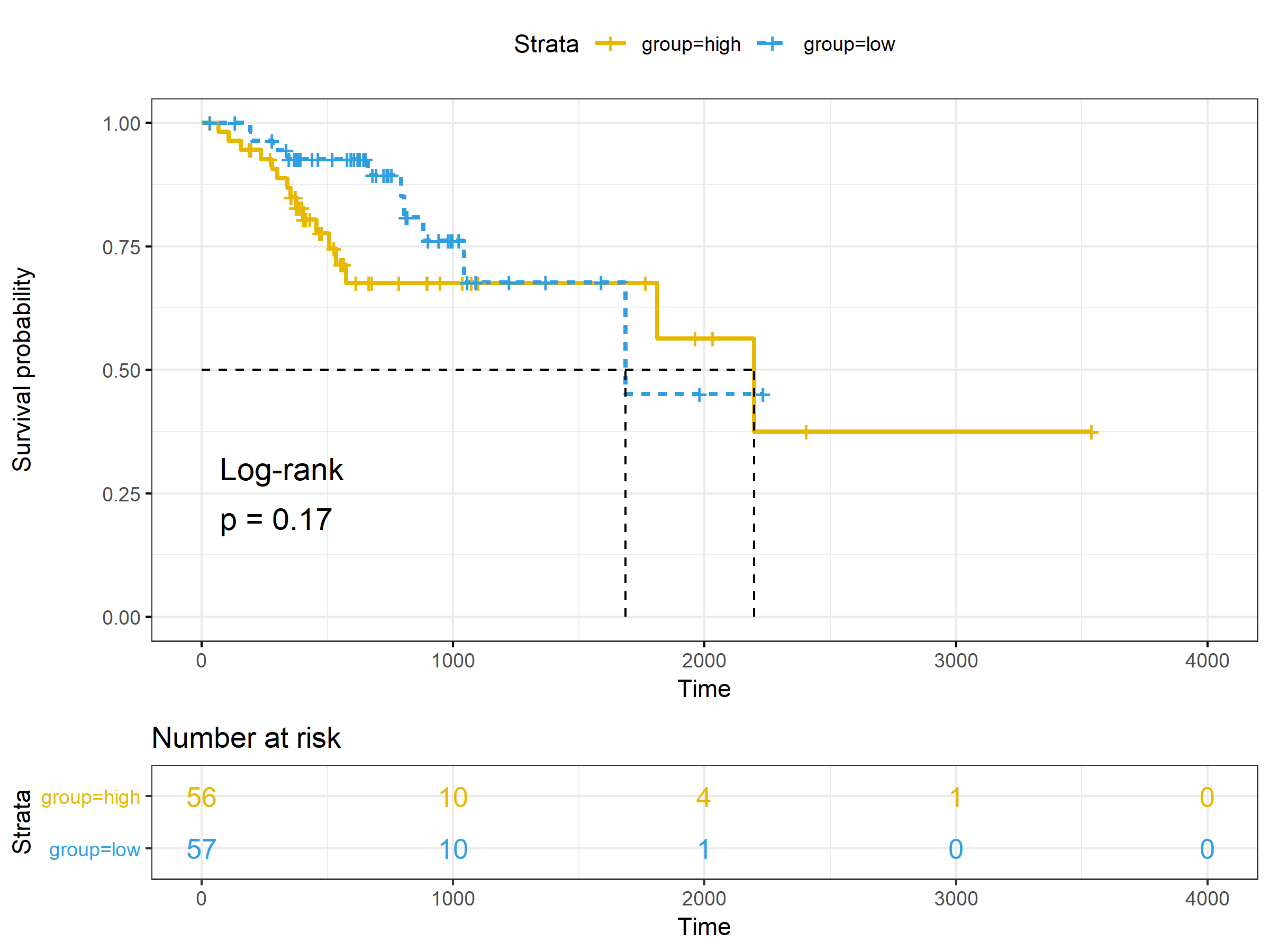

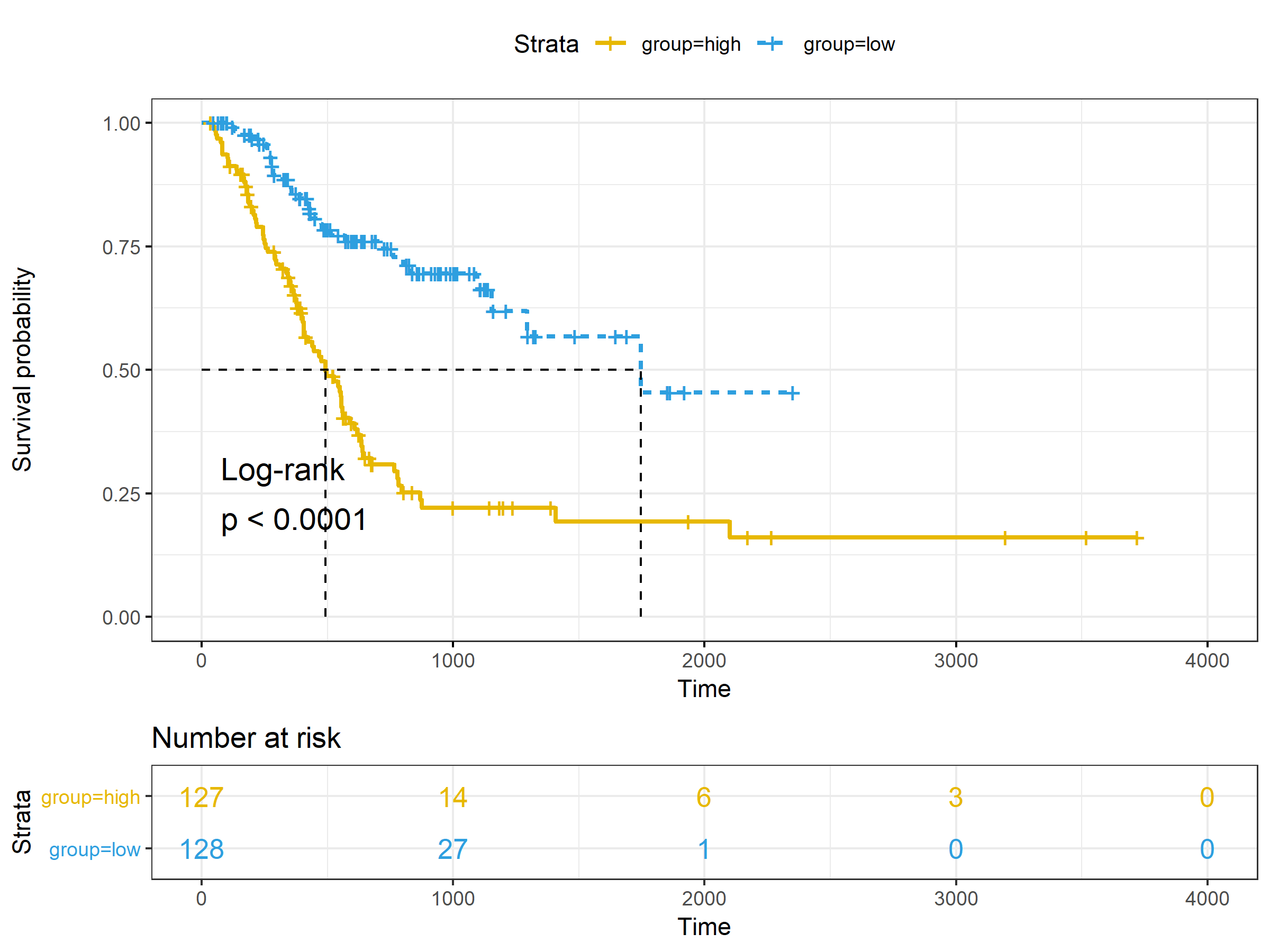


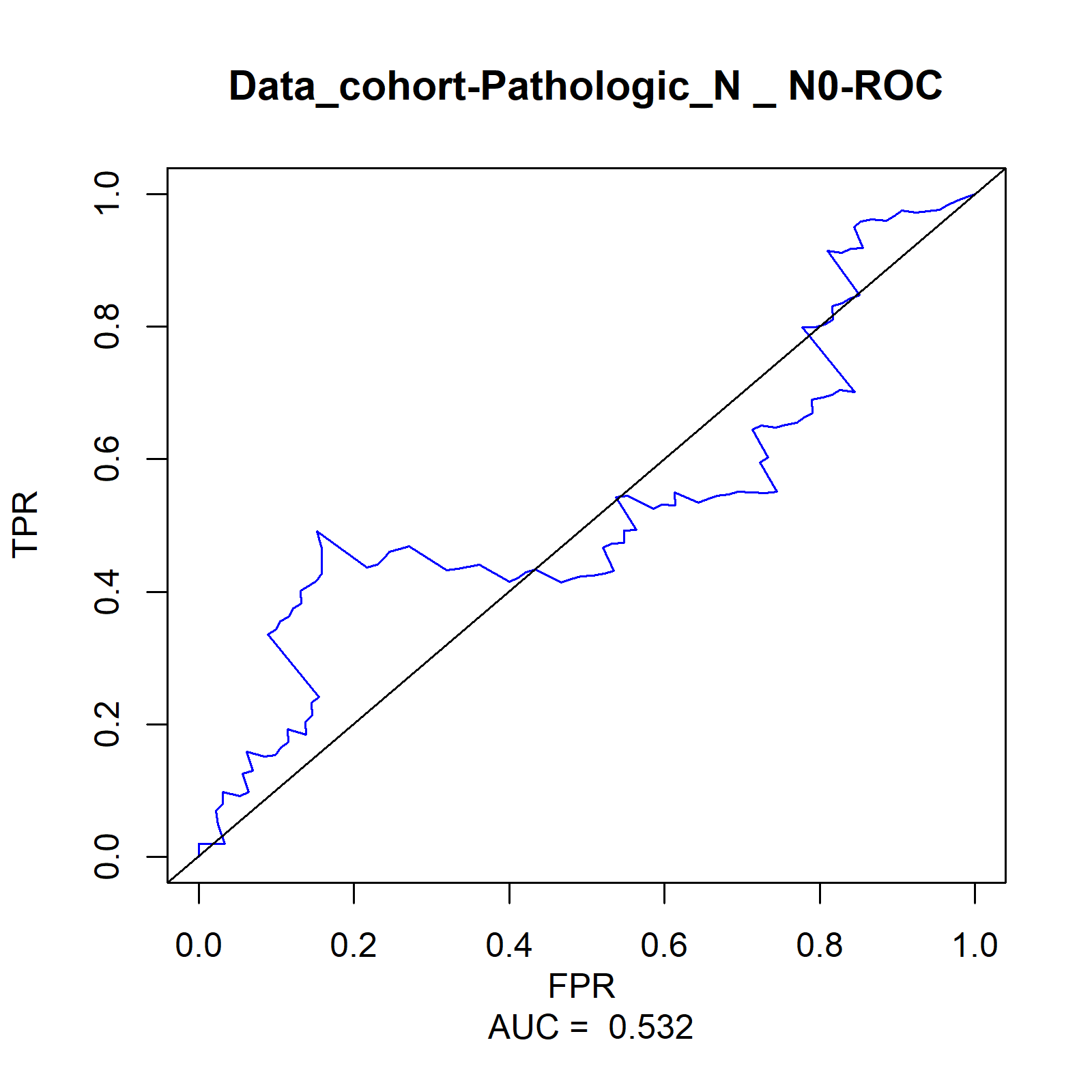

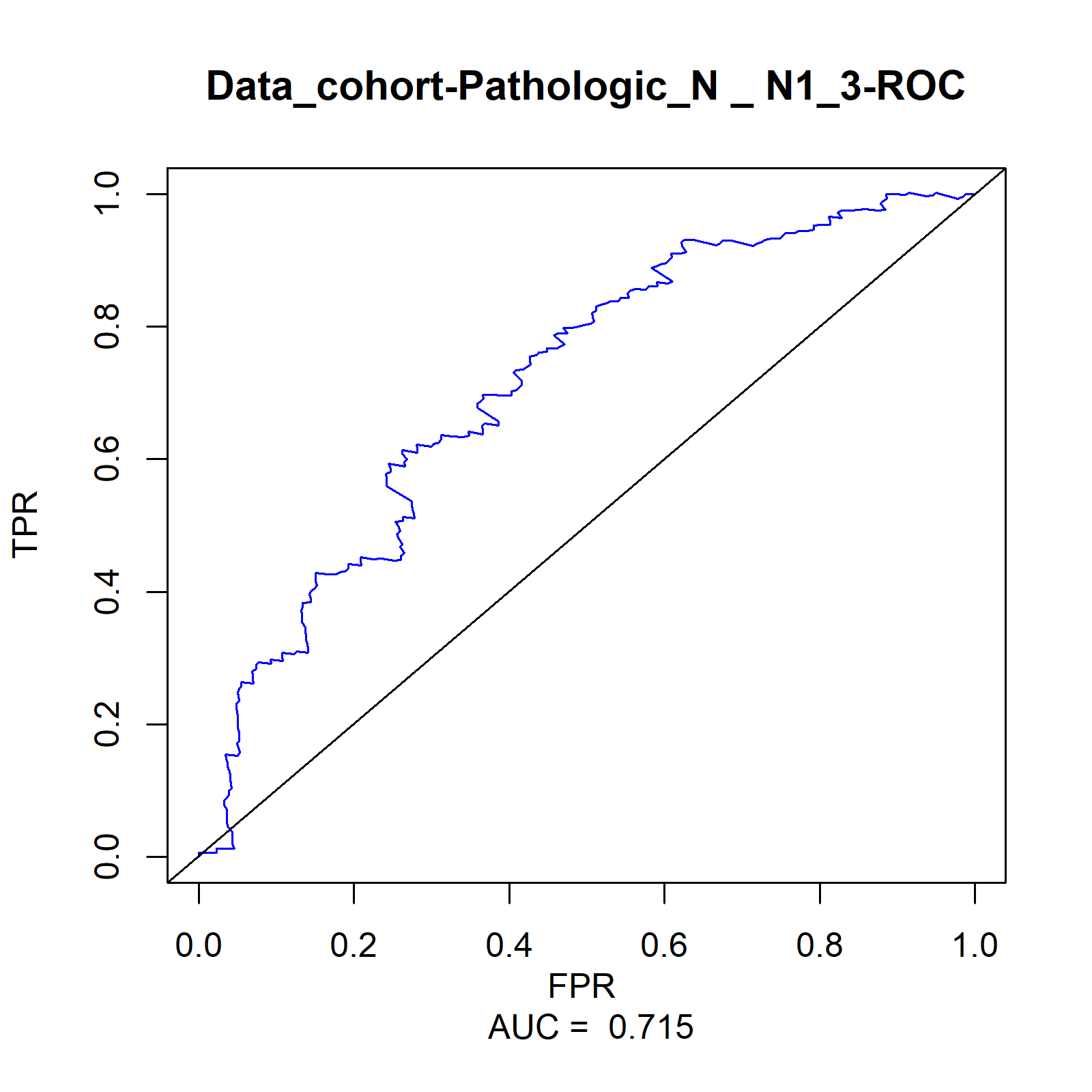


N_0_/N_1-3_


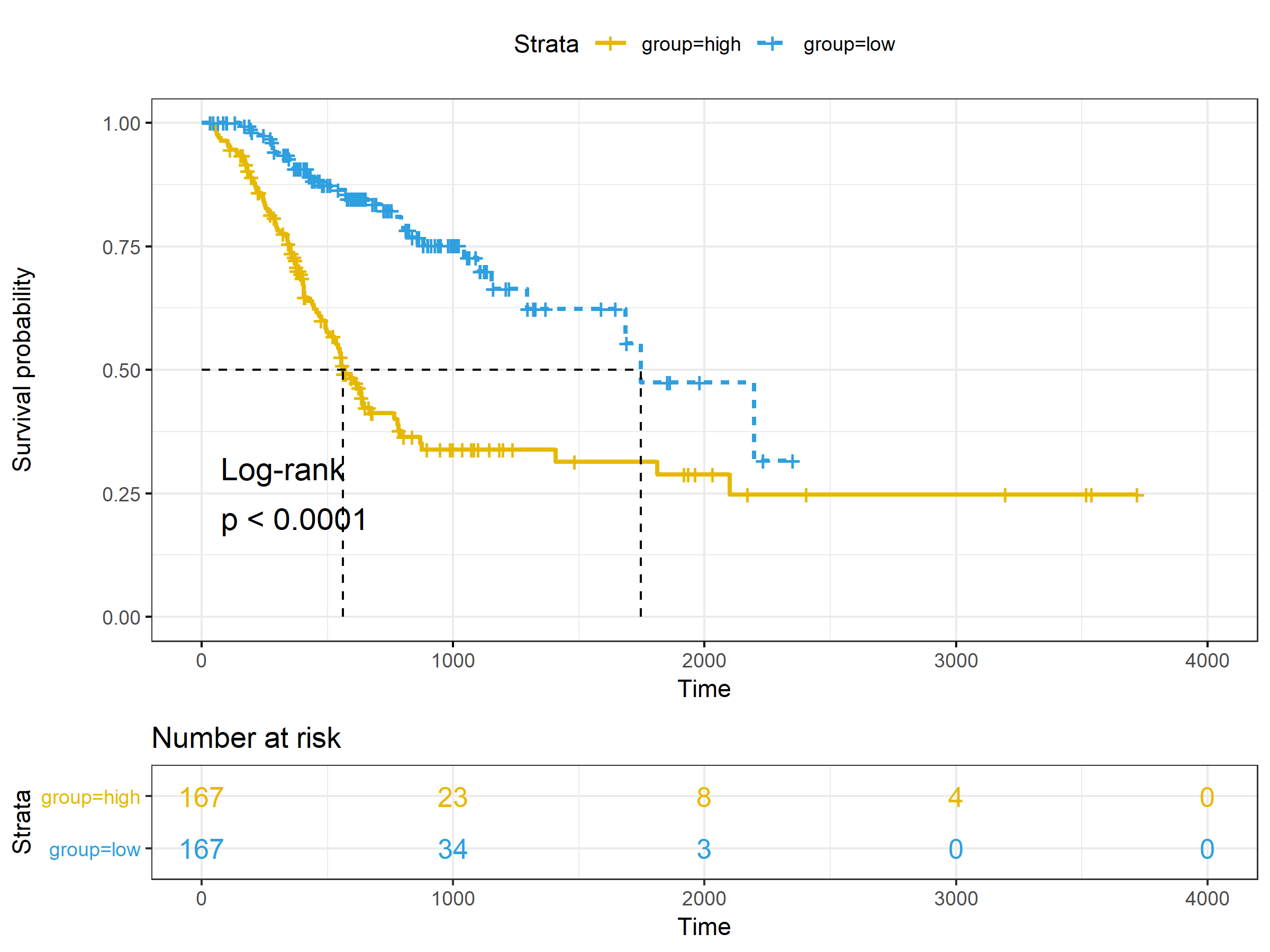

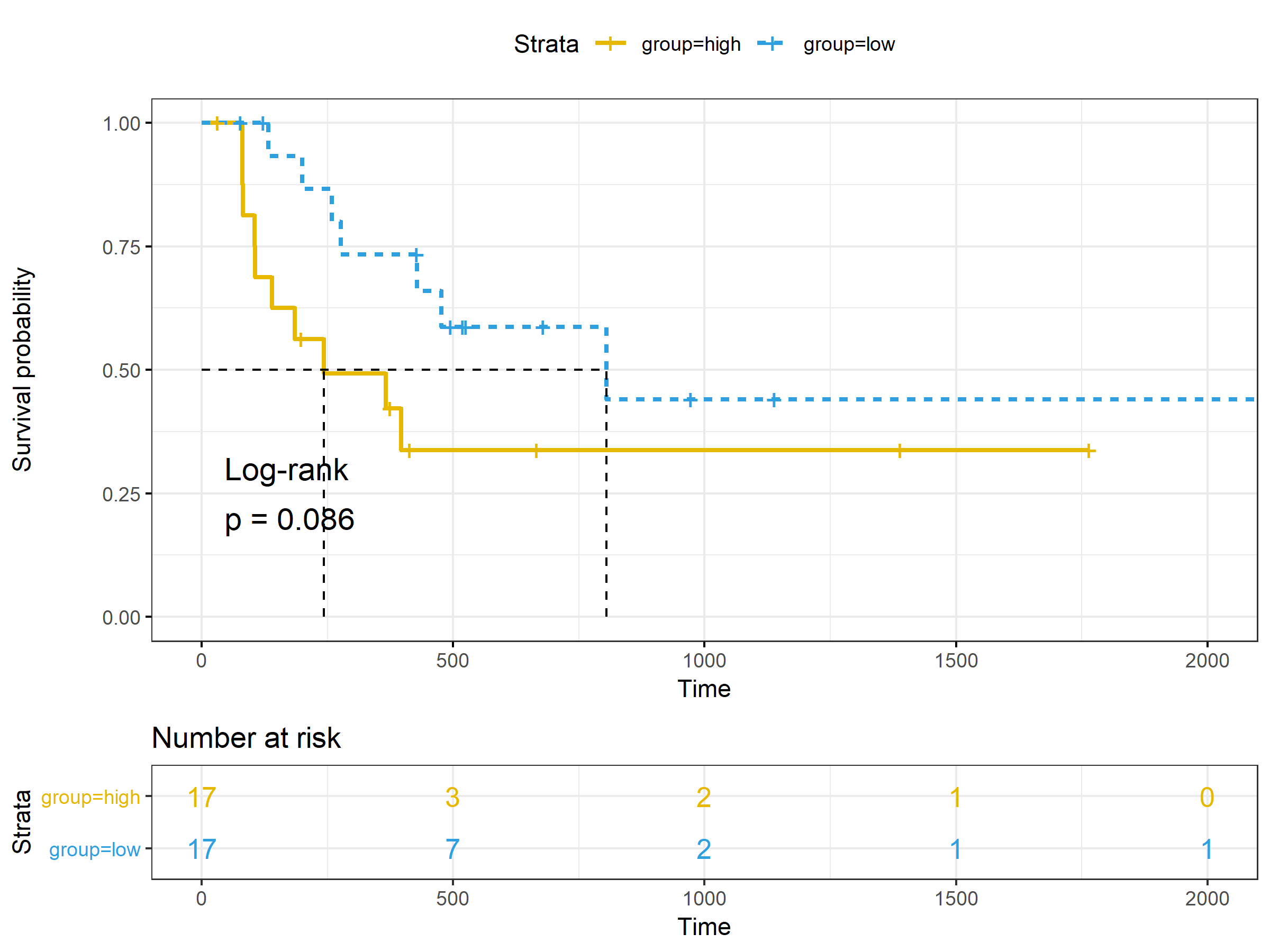


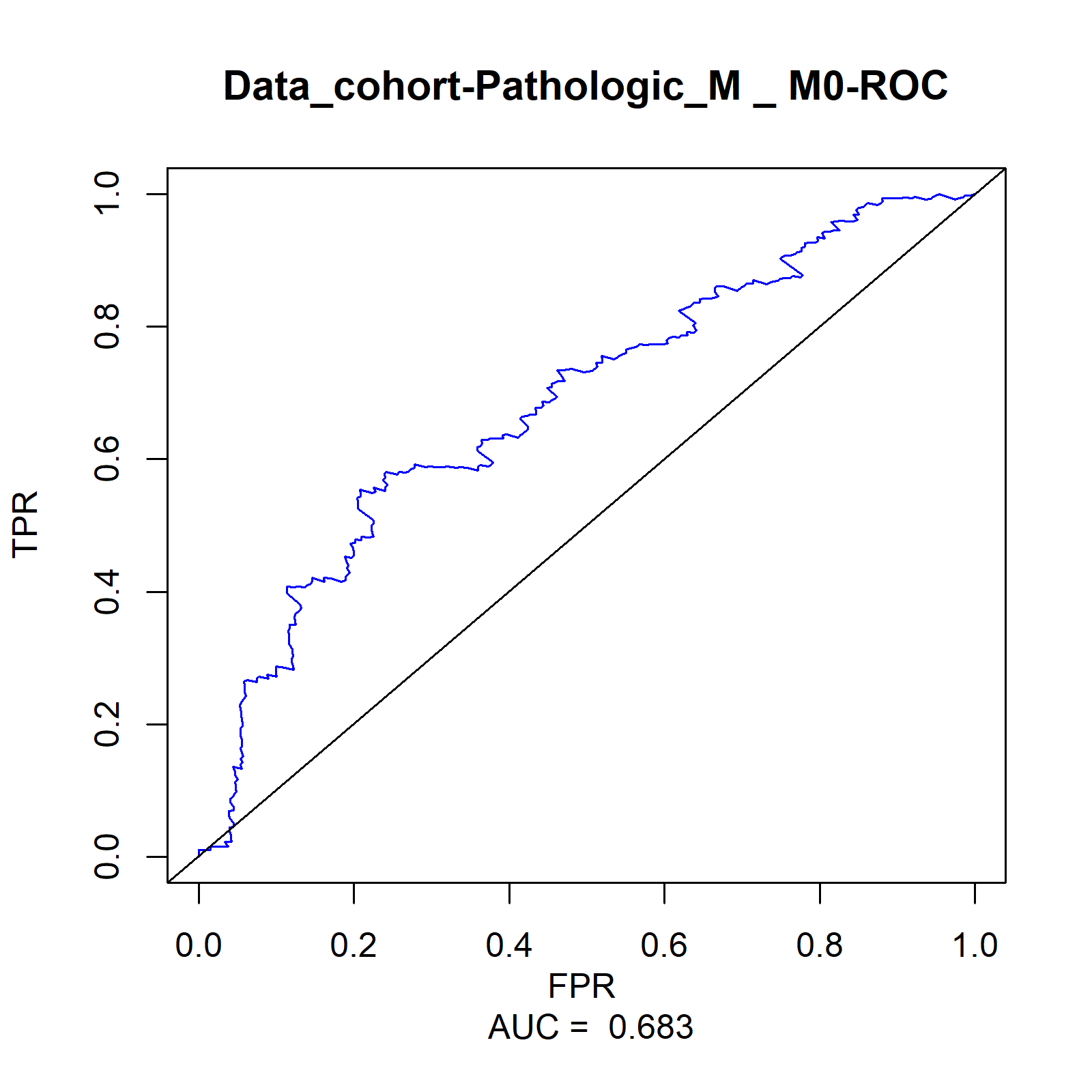

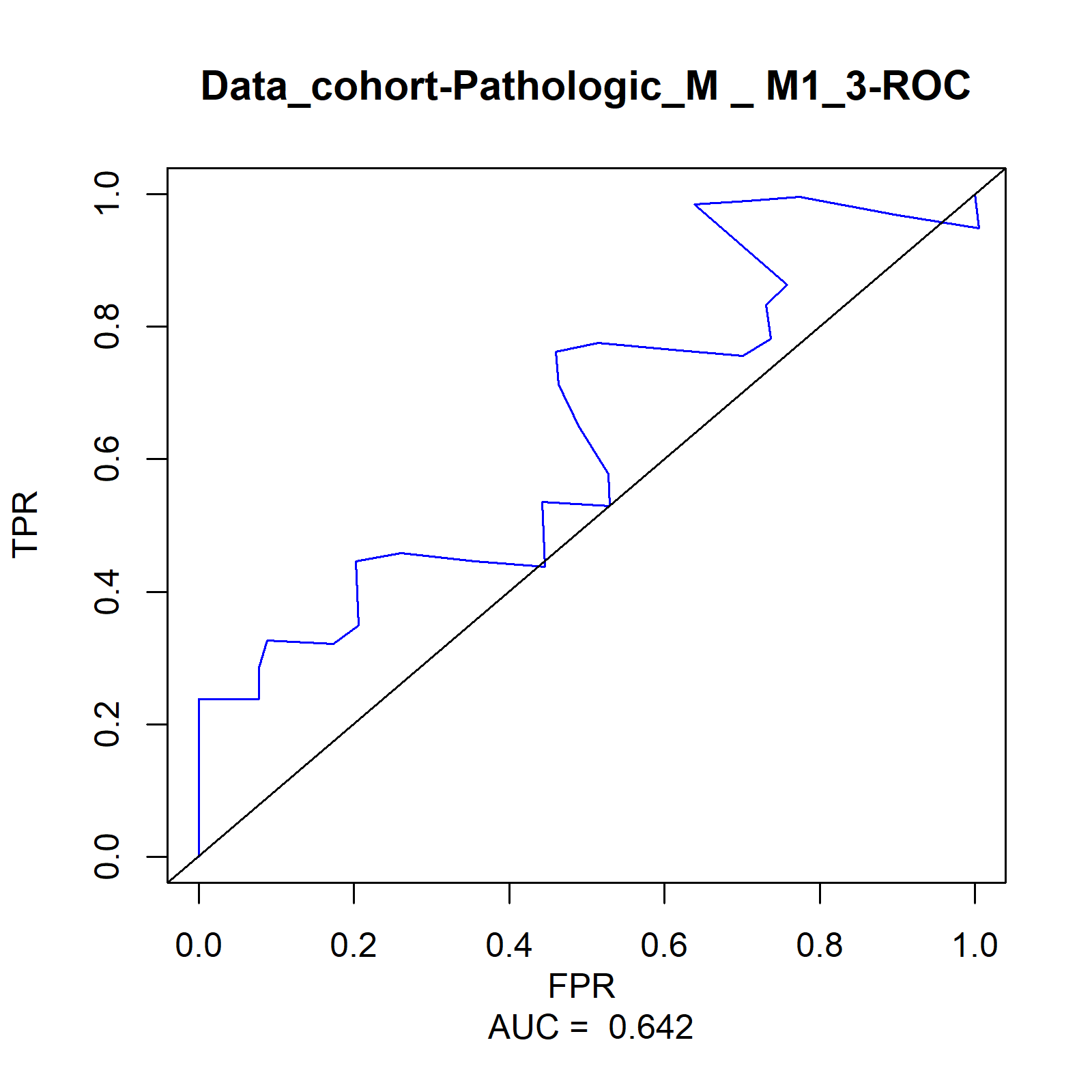


M_0_/M_1-3_

**Figure S3**. Kaplan-Meier and ROC analyses of GC patients in different cohorts regrouping by pathological stage (I-II and III-IV). **a.** Kaplan-Meier analysis with two-sided log-rank test was performed to assess the differences in OS between the low-risk and high-risk patients. **b.** ROC curves were to evaluate the predicting accuracy of four-DNA methylation biomarker for the prognosis of GC patients.


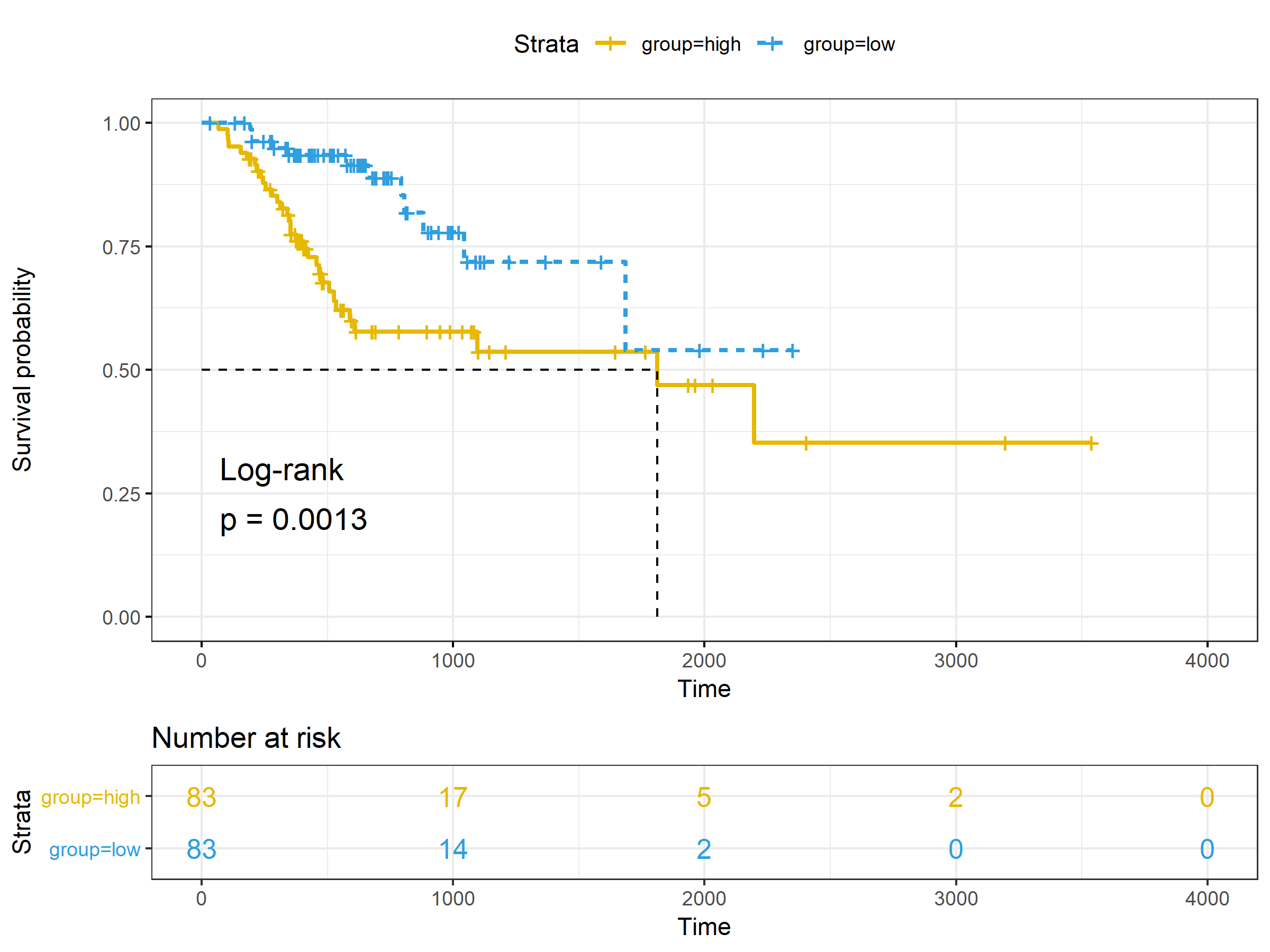

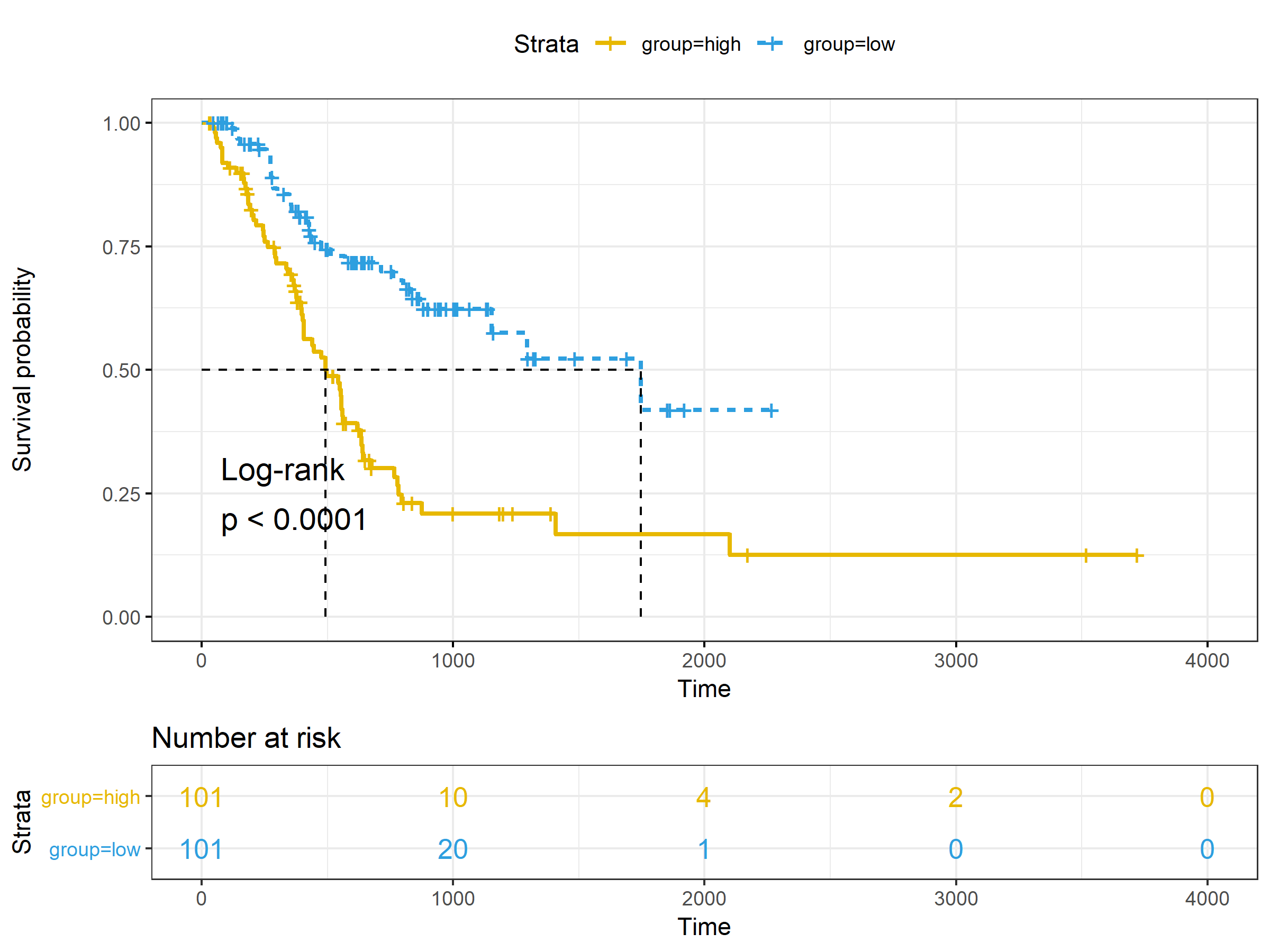


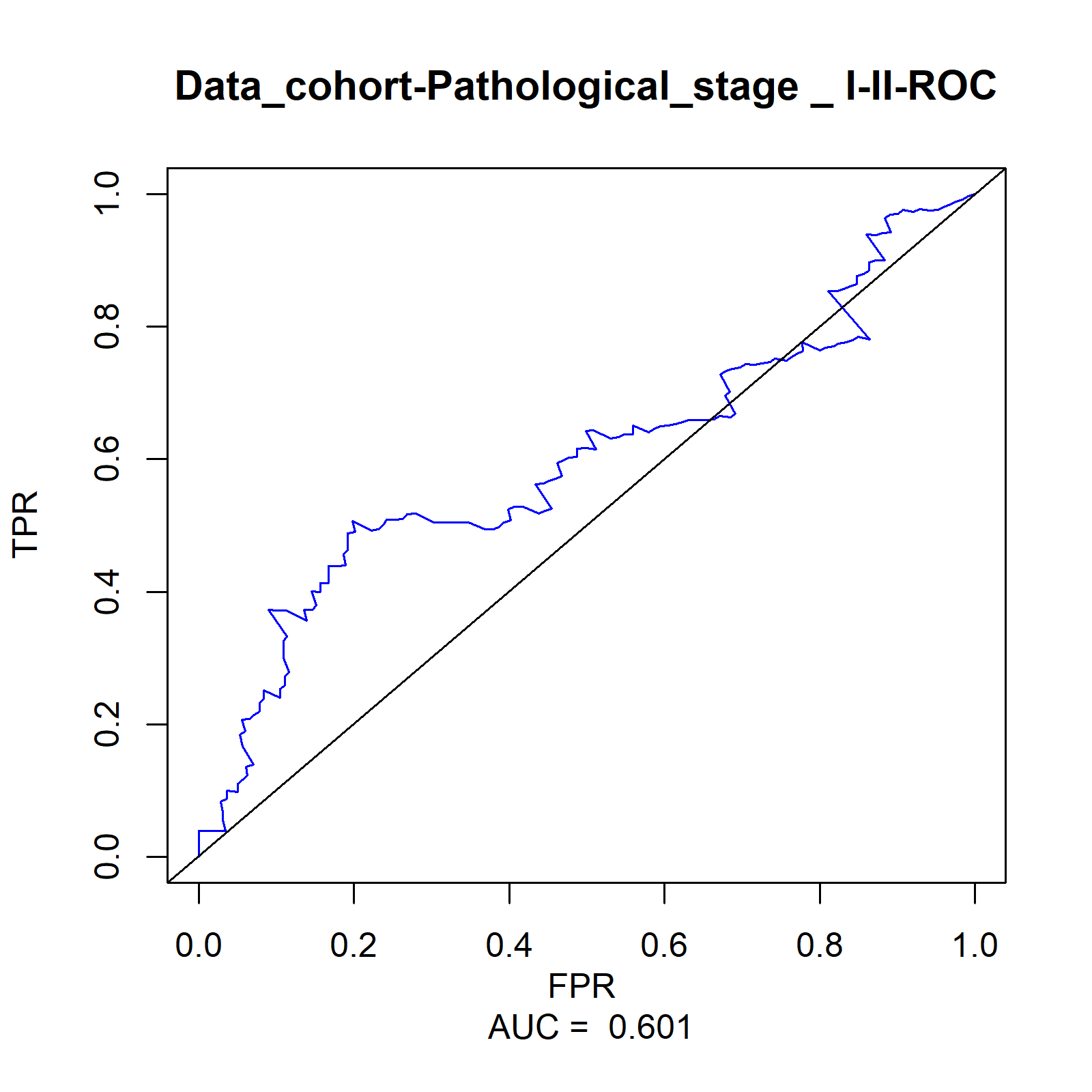

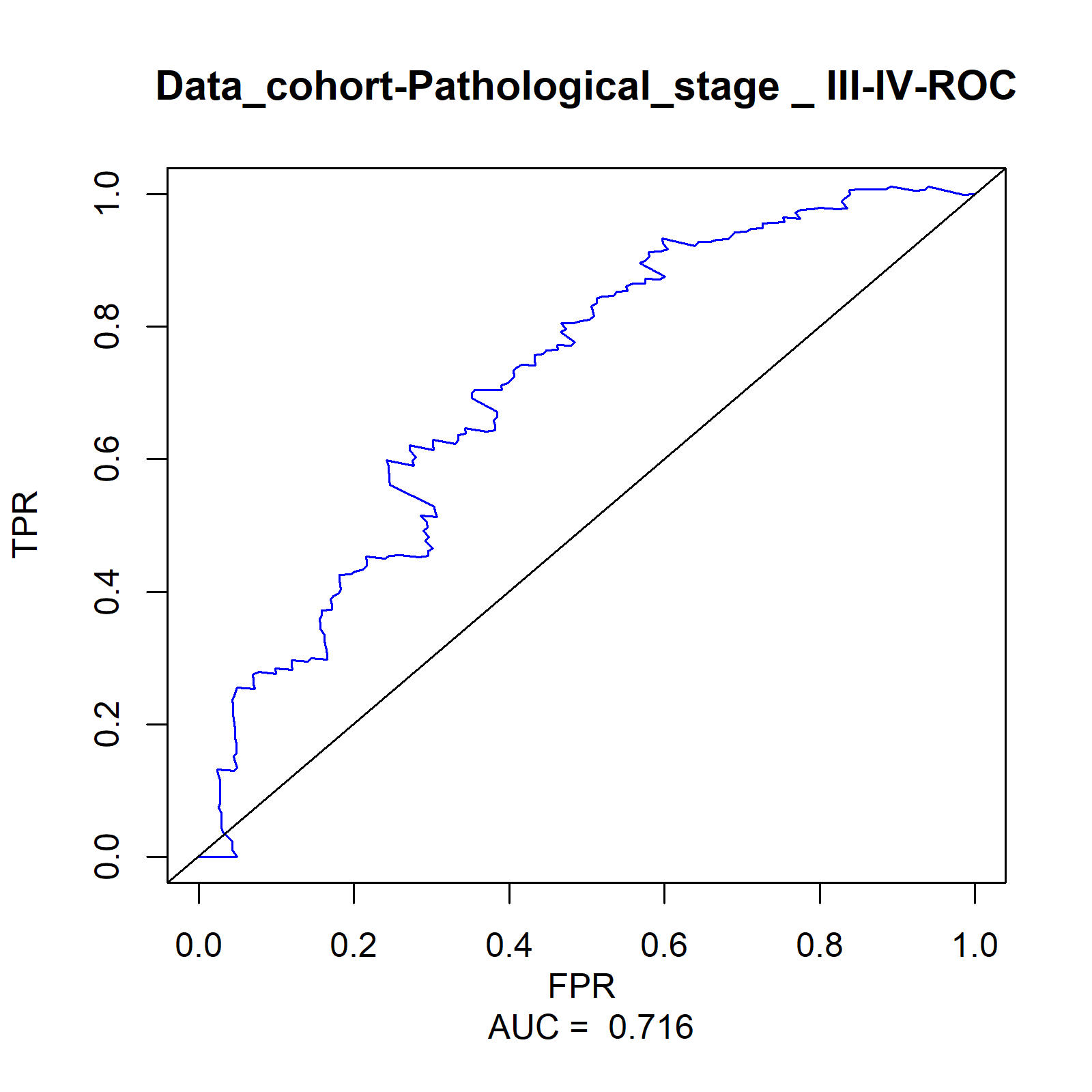


Pathological stage (I-II and III-IV)

**Figure S4**. Kaplan-Meier and ROC analyses of GC patients in different cohorts regrouping by histologic grade of GC. **a.** Kaplan-Meier analysis with two-sided log-rank test was performed to assess the differences in OS between the low-risk and high-risk patients (G1-2/G3-4). **b.** ROC curves were to evaluate the predicting accuracy of four-DNA methylation biomarker for the prognosis of GC patients.


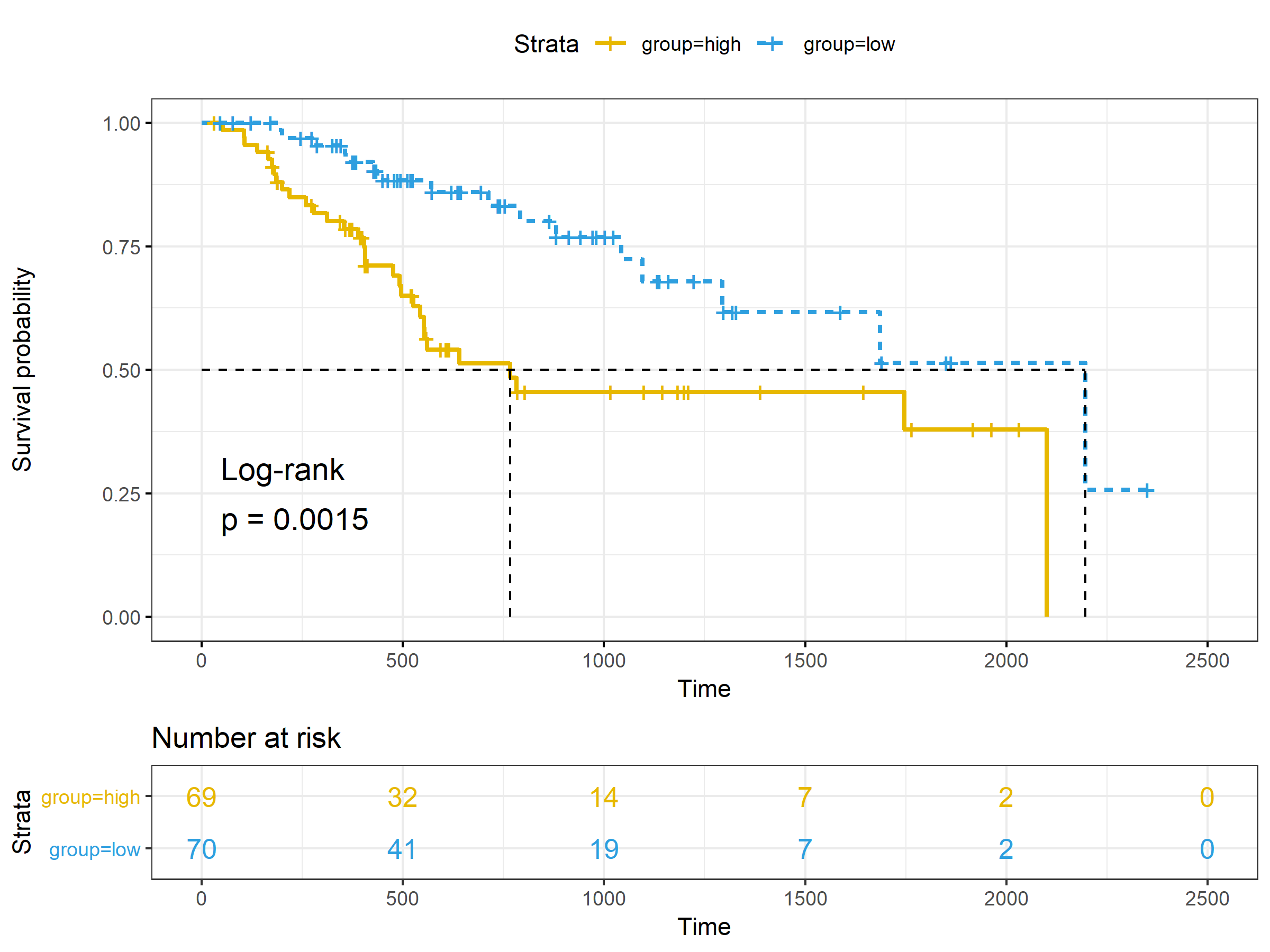

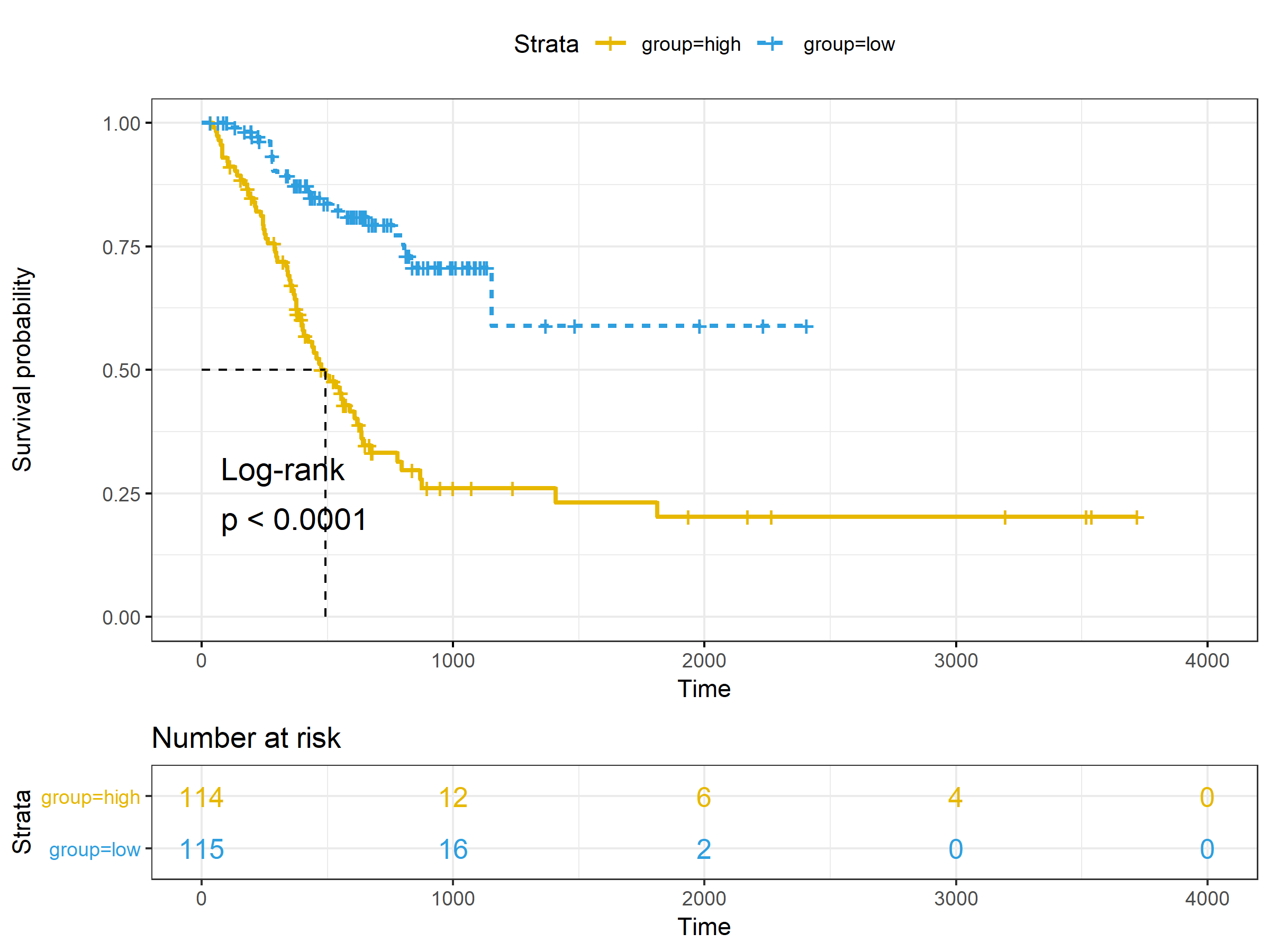


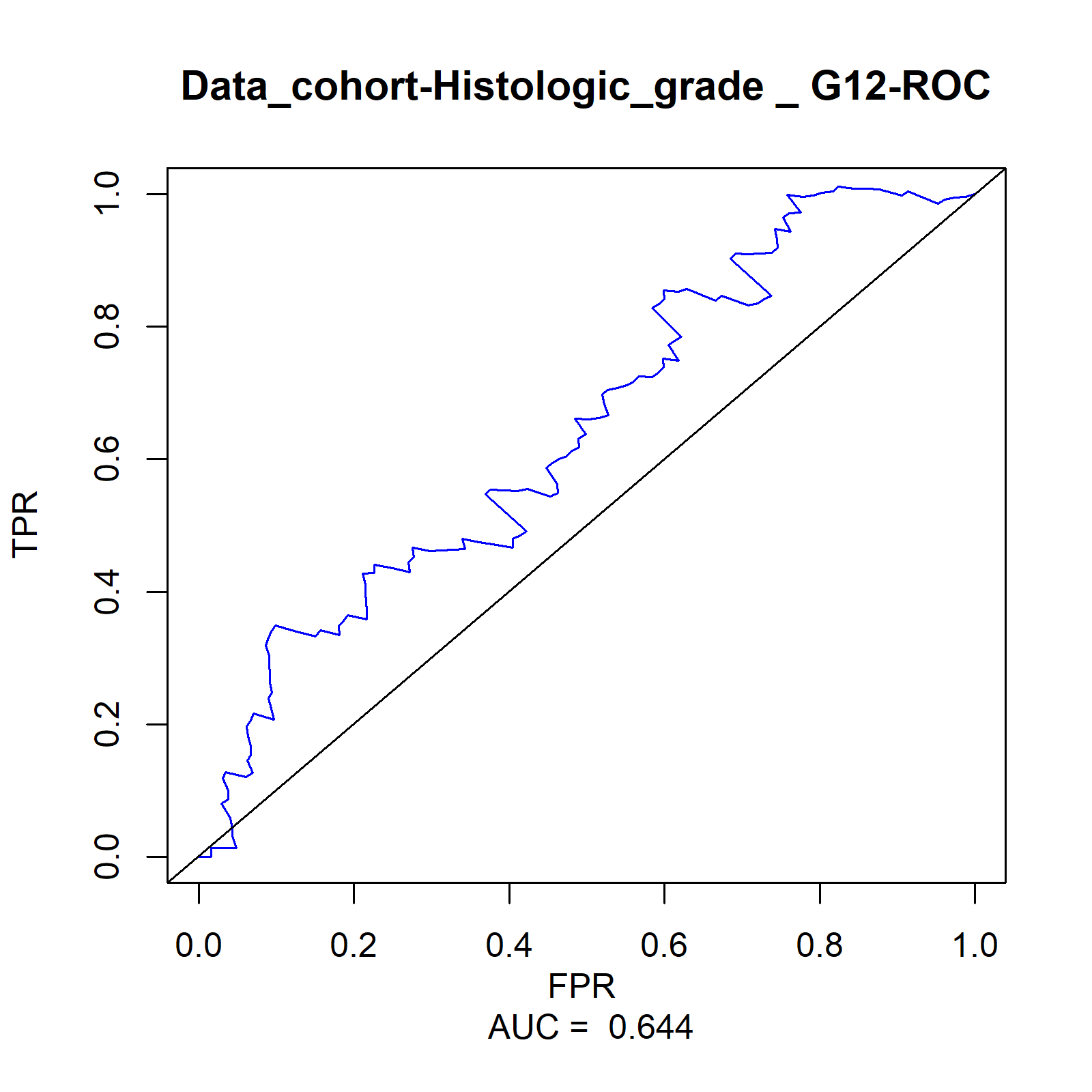

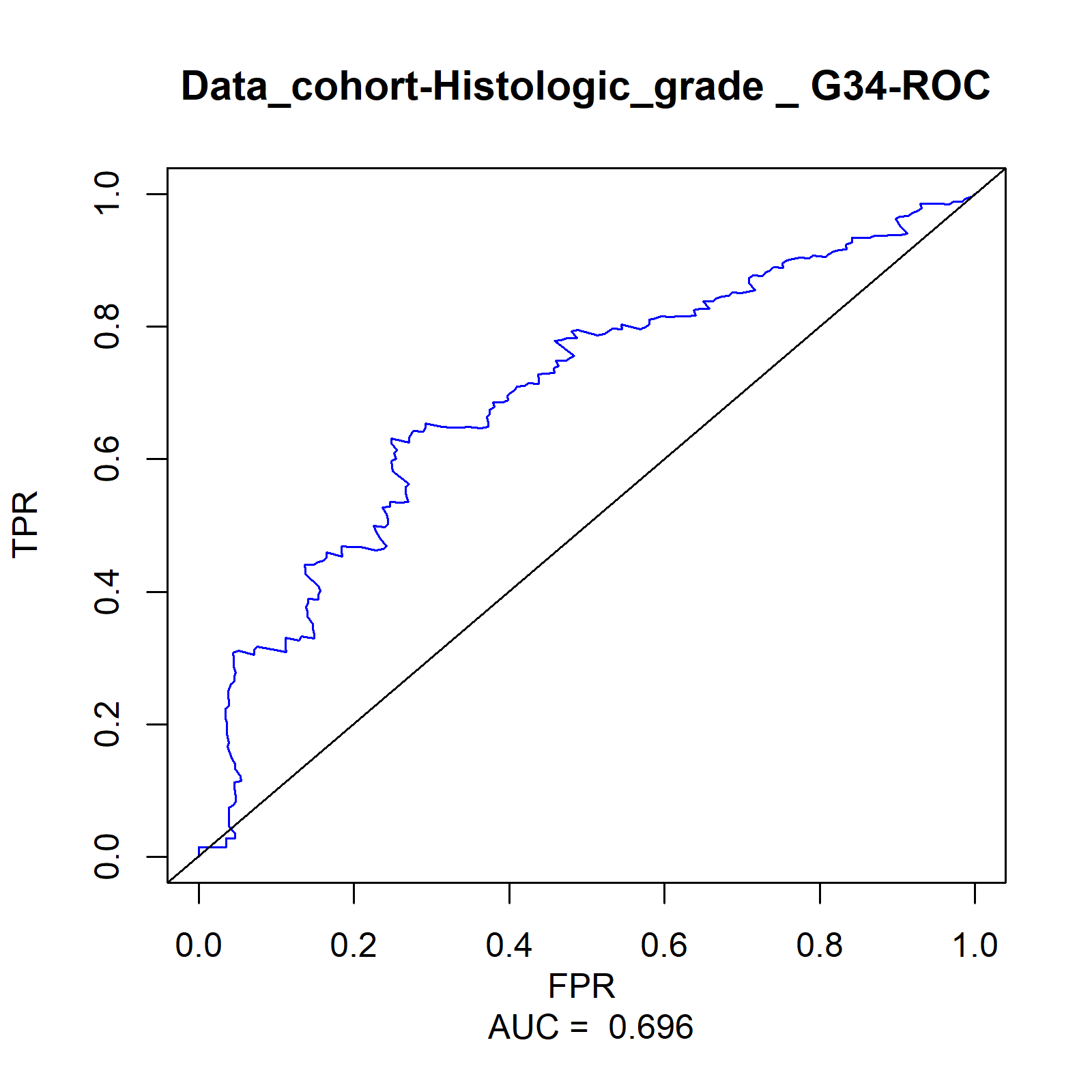


Histologic grade (G1-2/G3-4)

**Figure S5**. Kaplan-Meier and ROC analyses of GC patients in different cohorts regrouping on the anatomical region of GC: antrum, cardia, fudus, gastroesophageal junction (GEJ) and unclear type. **a.** Kaplan–Meier analysis with two-sided log-rank test was performed to assess the differences in OS between the low-risk and high-risk patients. **b.** ROC curves were to evaluate the predicting accuracy of four-DNA methylation biomarker for the prognosis of GC patients.


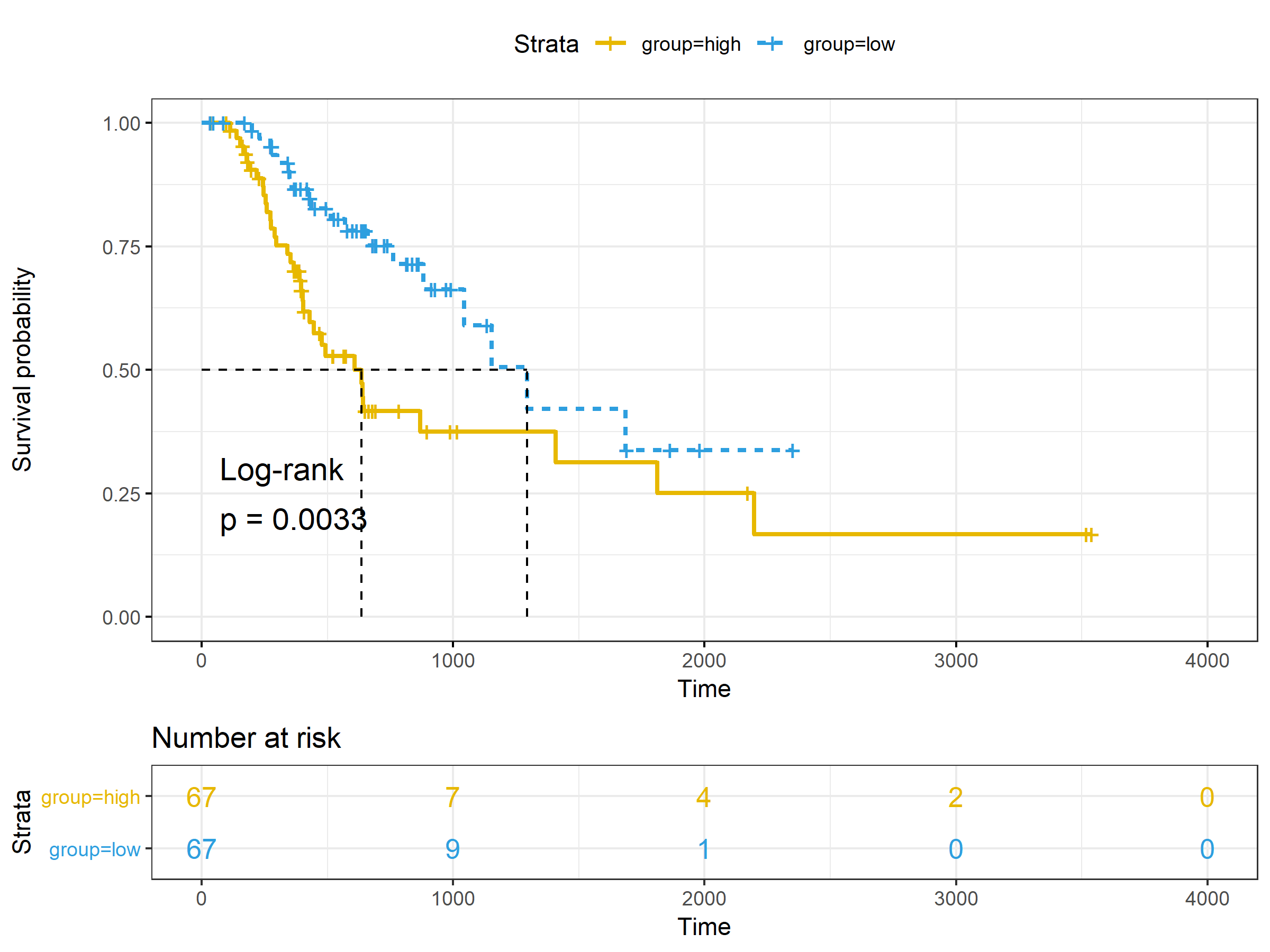

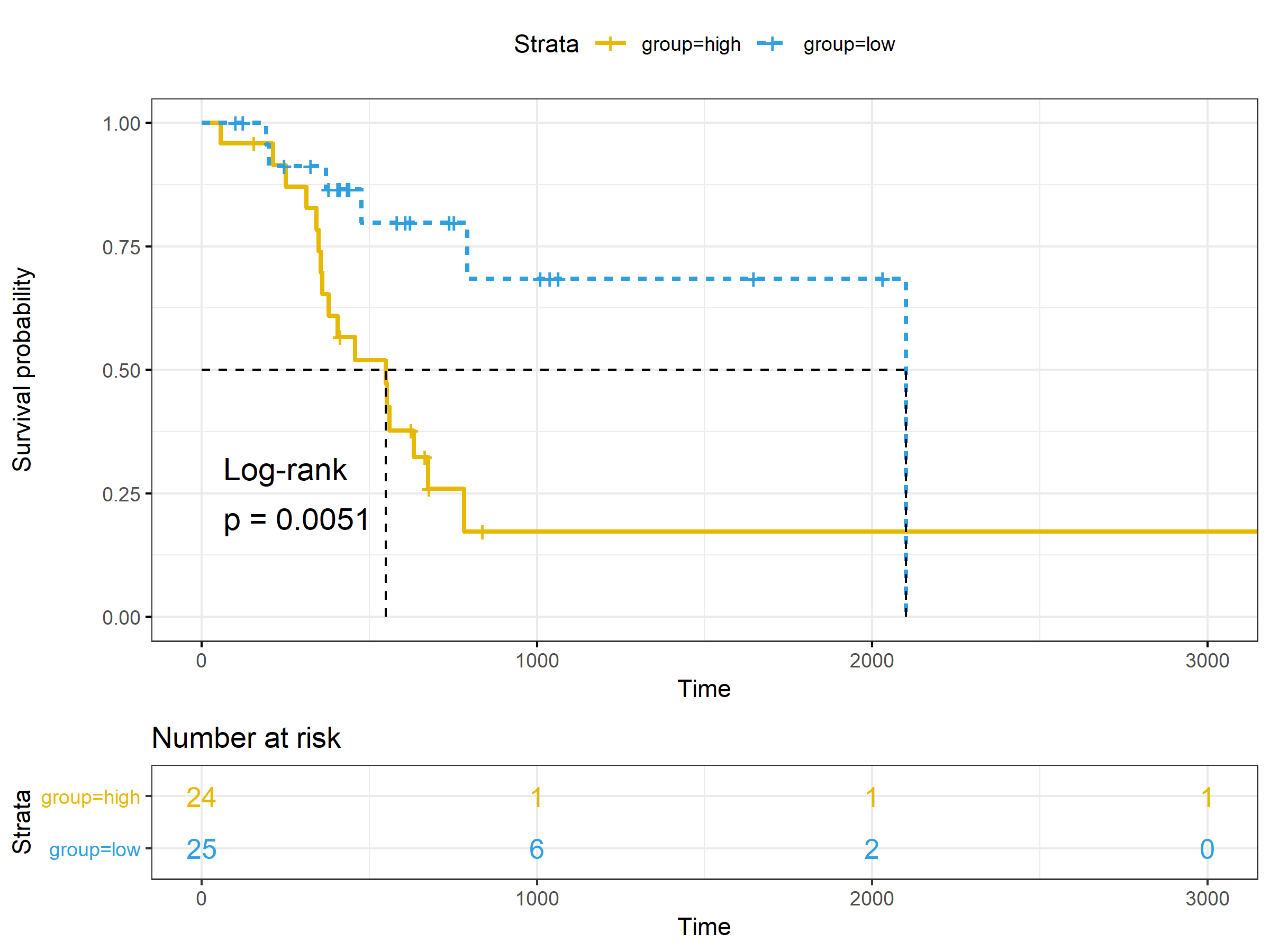


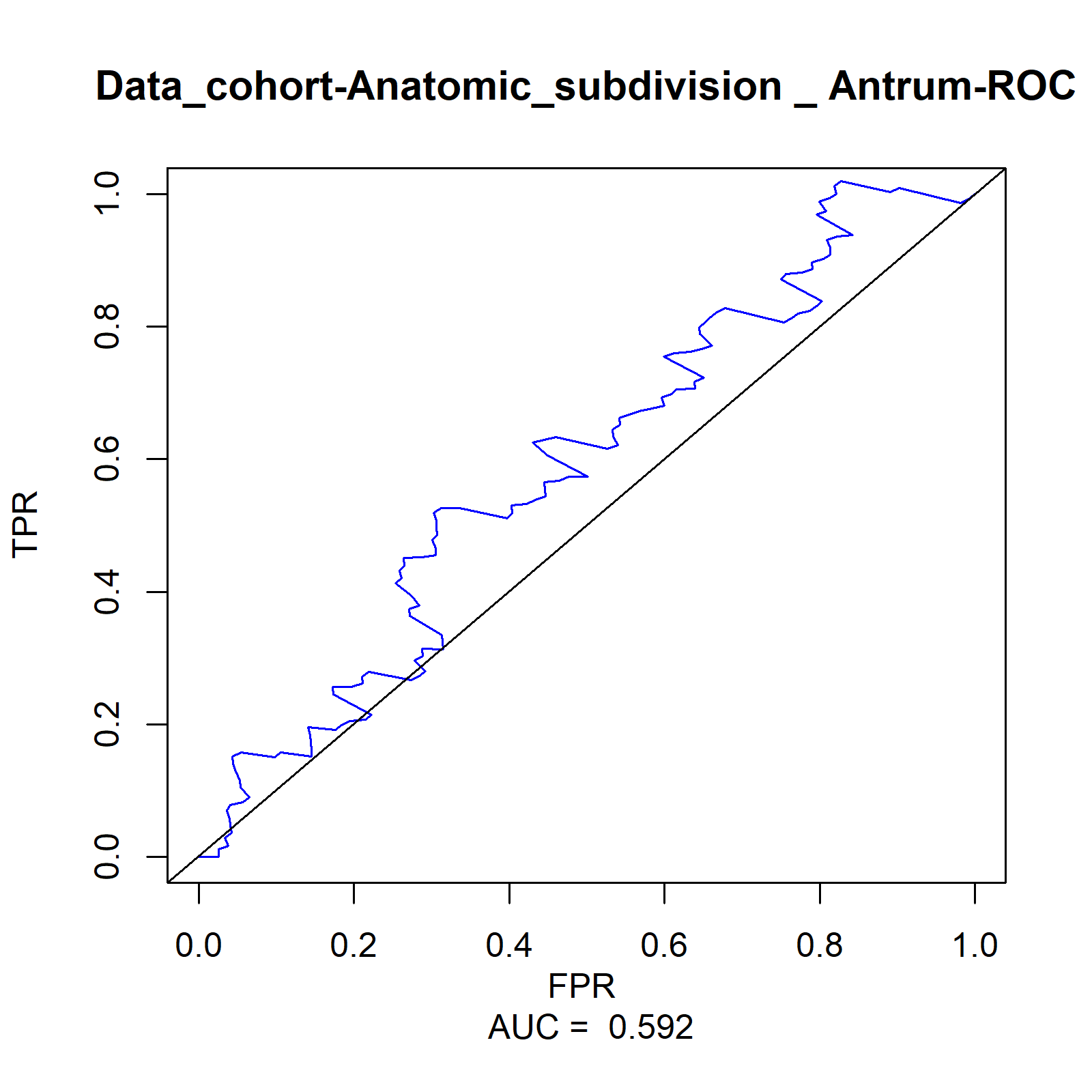

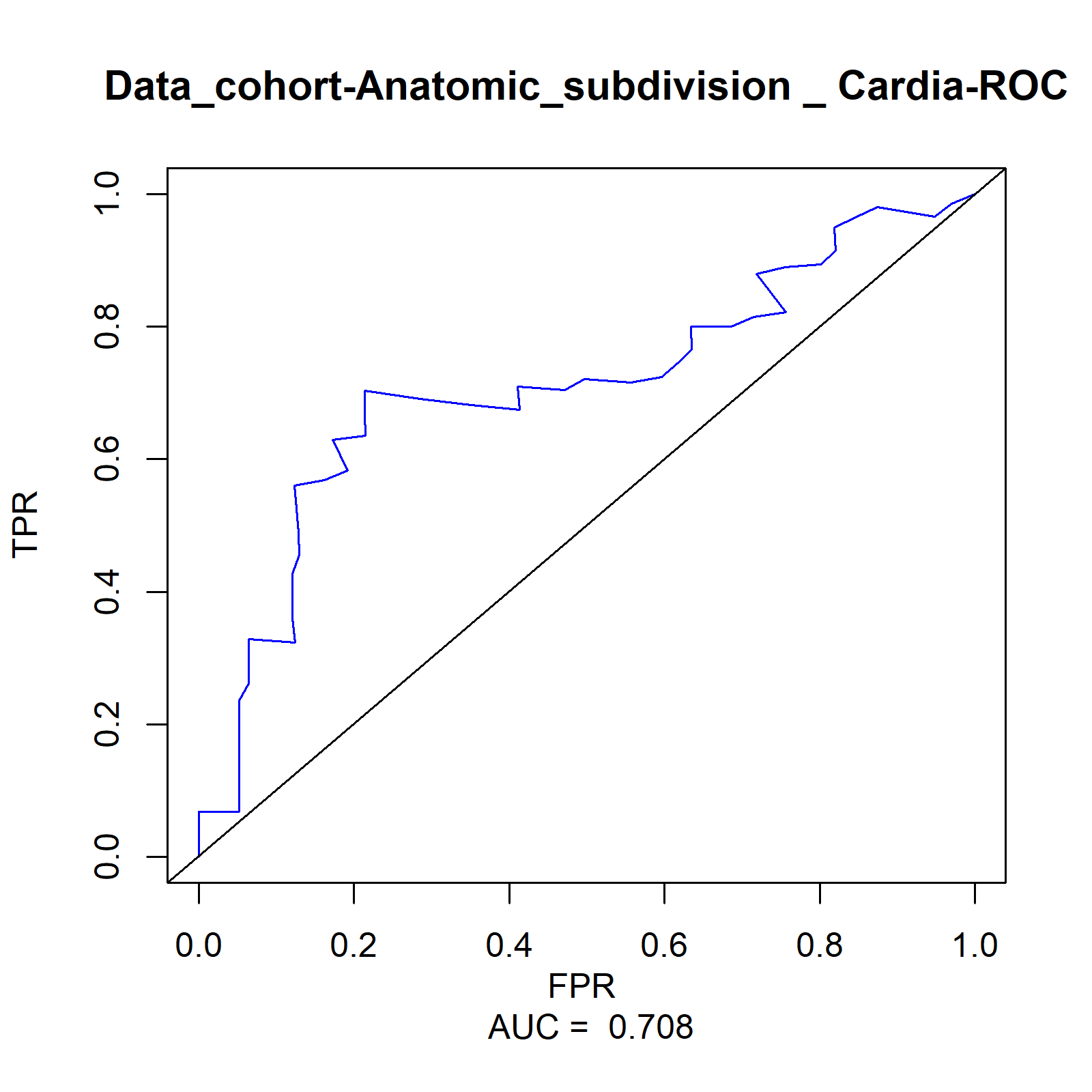


Antrum and cardia


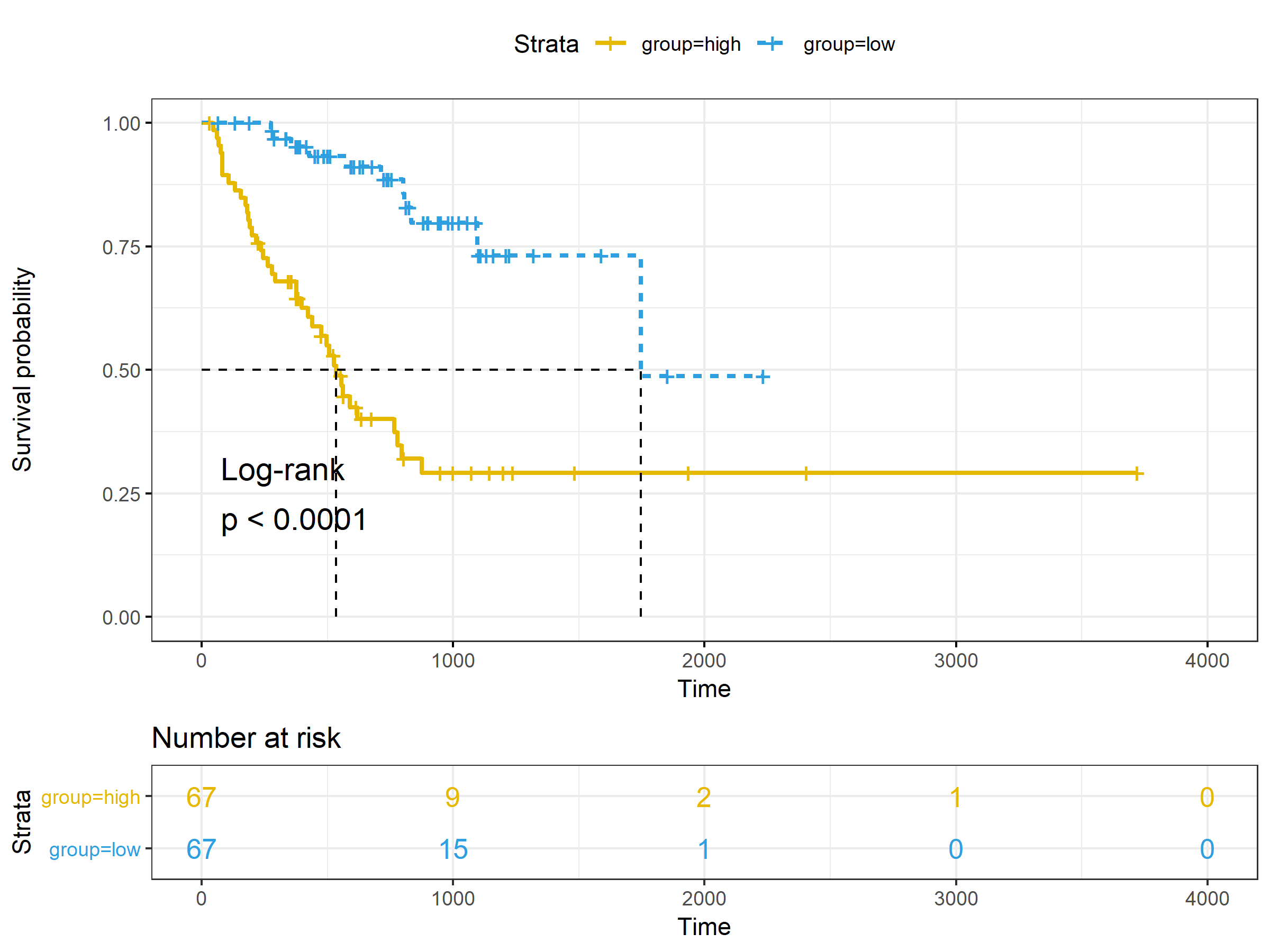

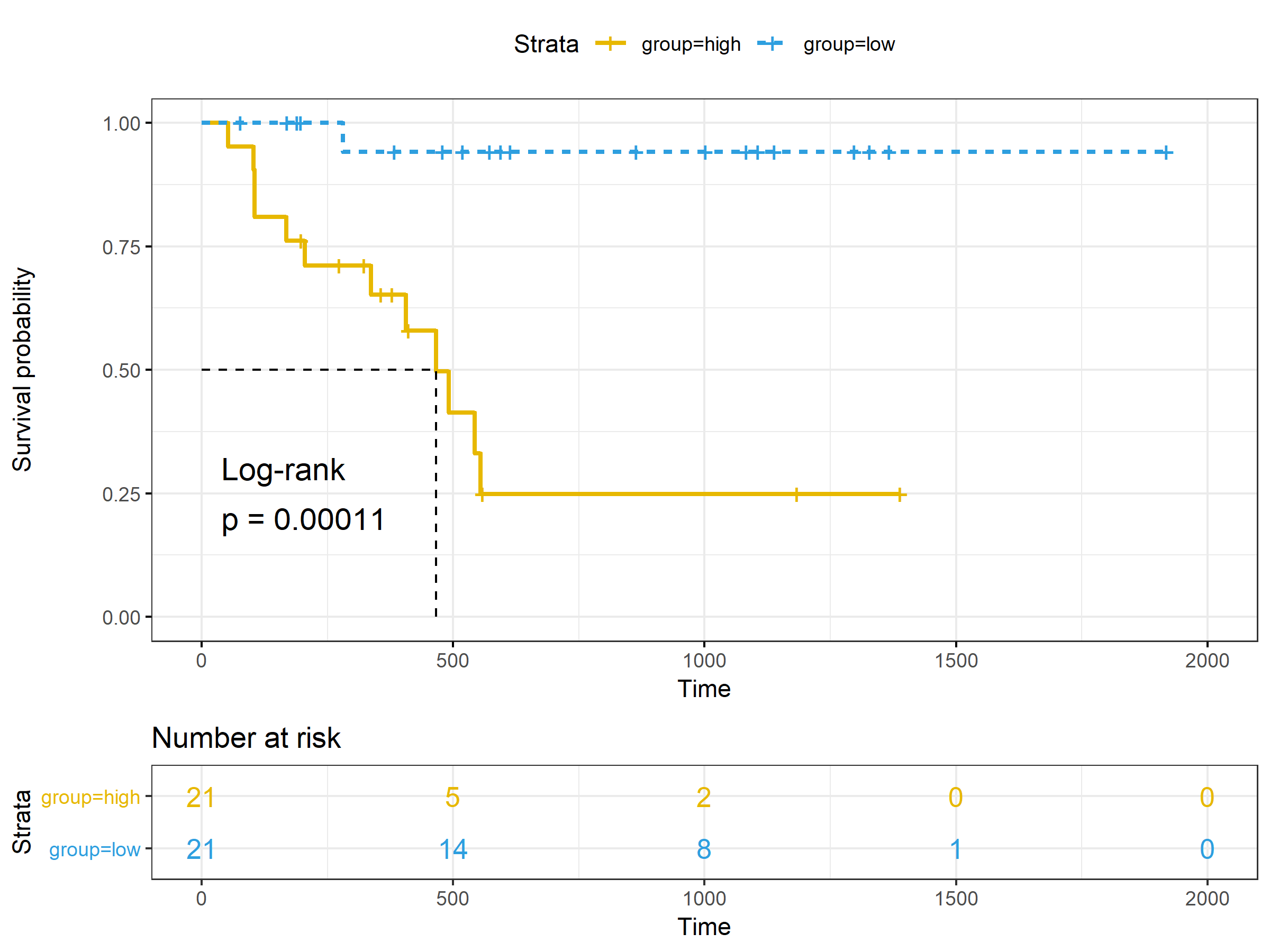


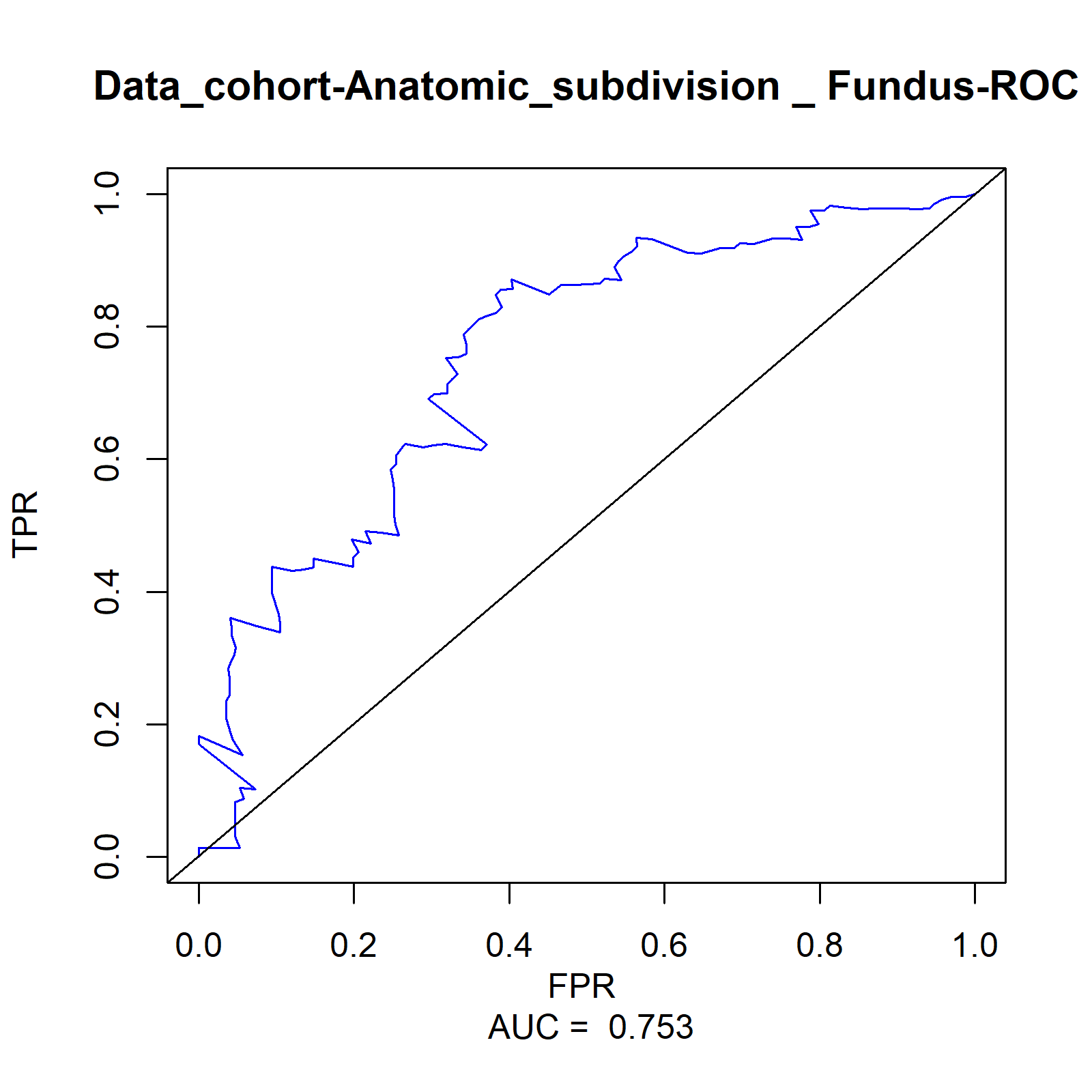

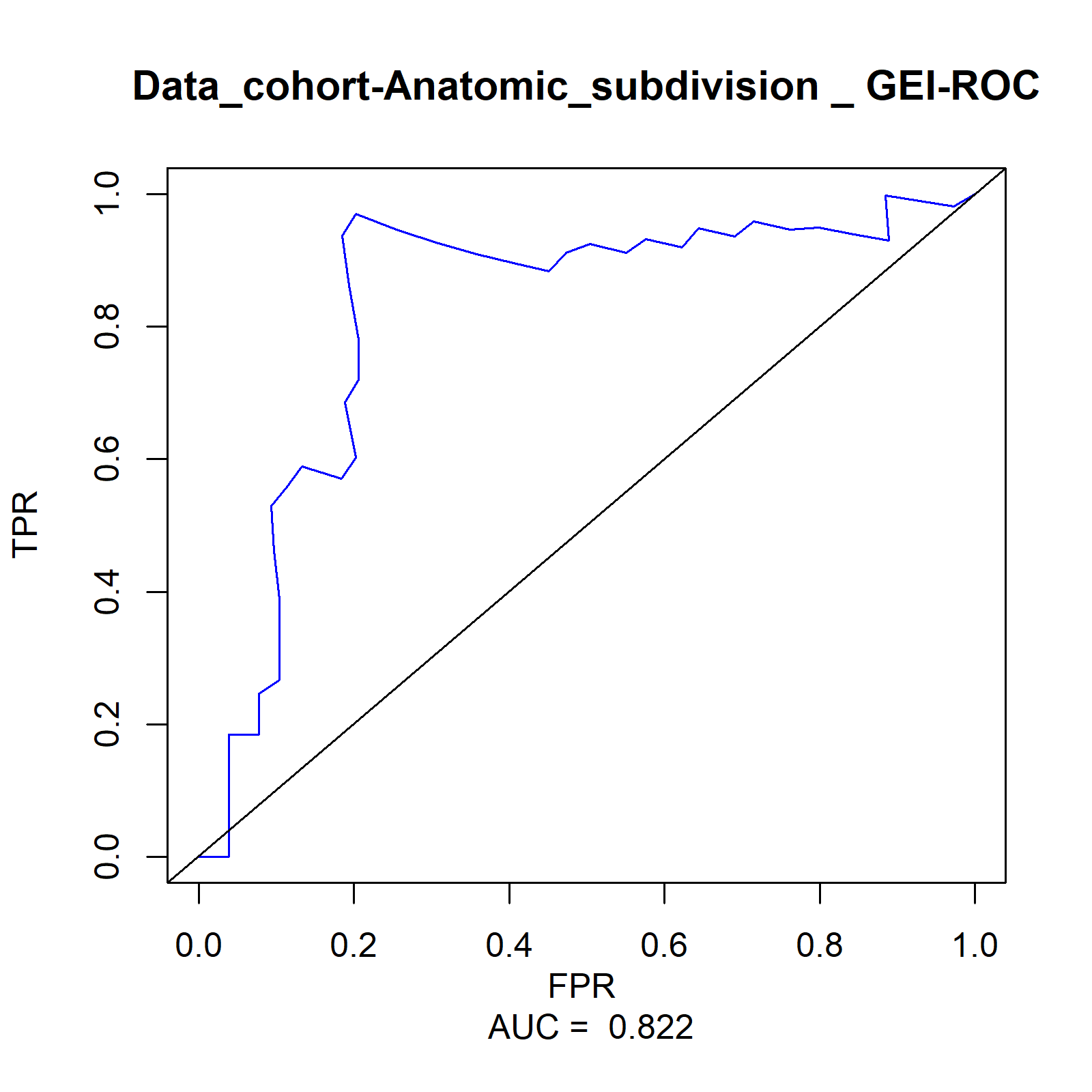


Fudus and GEJ


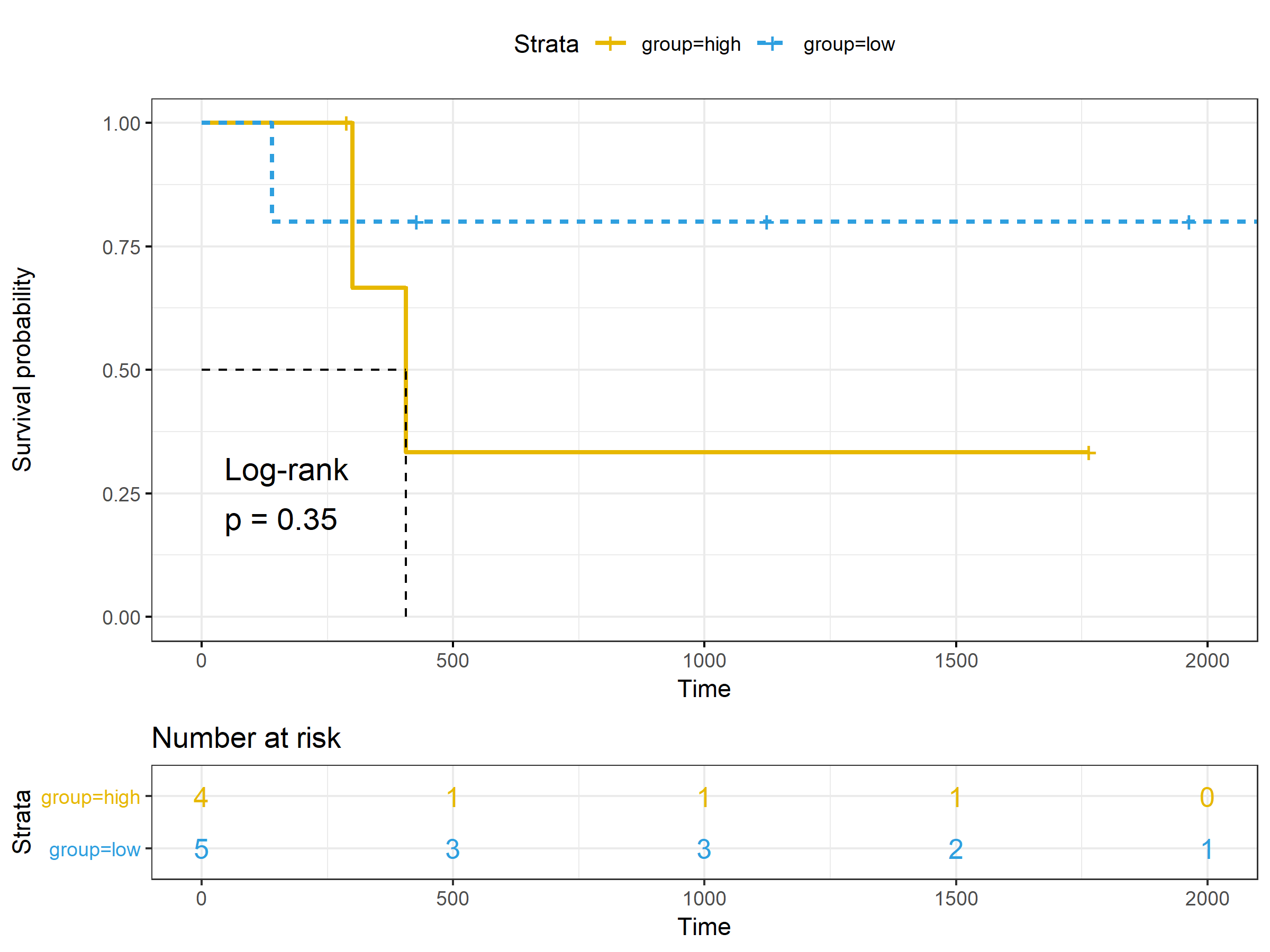

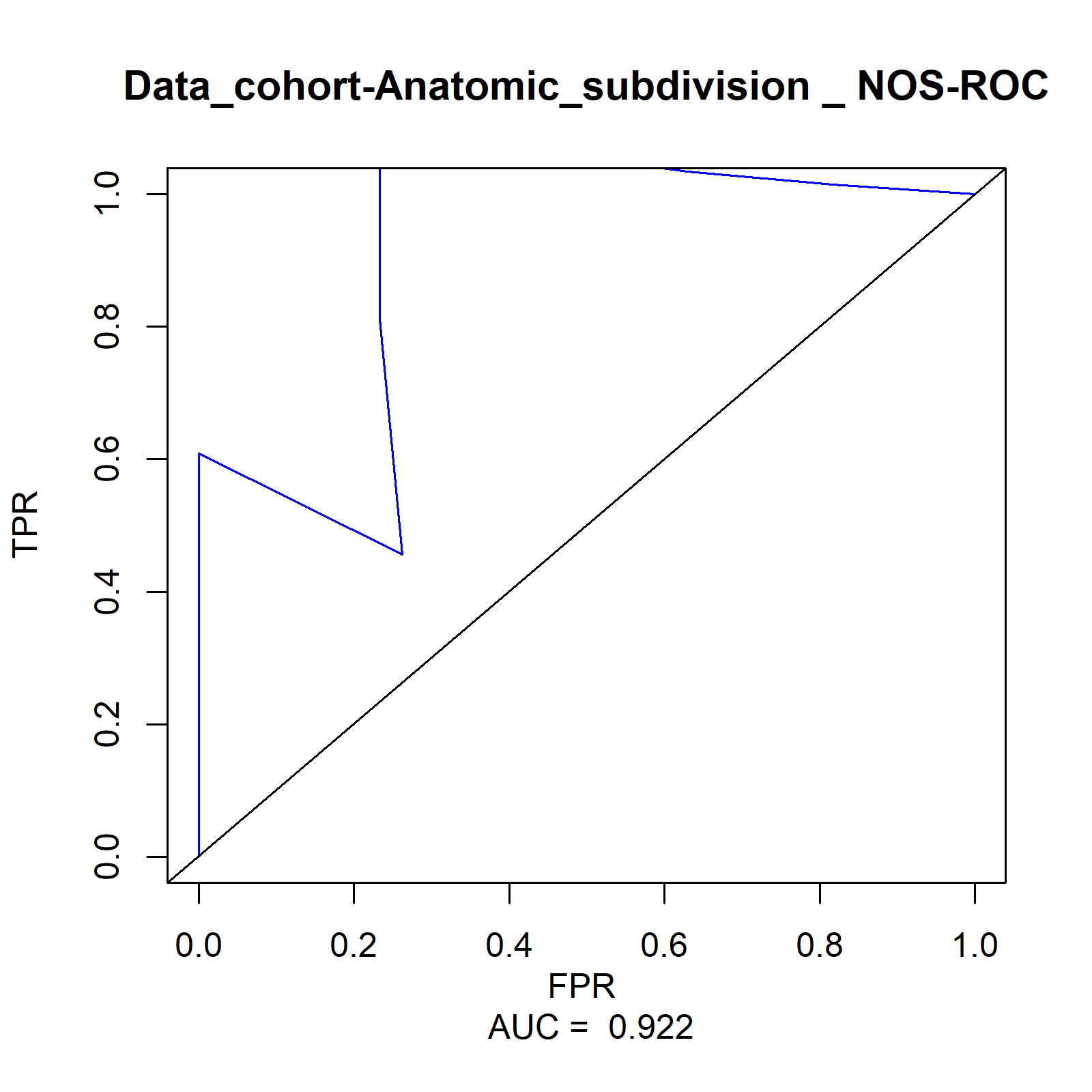


Unclear type

**Table S1** Results of comparison of different prognostic biomarkers

| Biomarker | AUC | CI95% | P value | Roc test |
| --- | --- | --- | --- | --- |
| four-DNA methylation | 0.71 | 0.66-0.77 | 2.23E-14 | 1 |
| Seven-miRNA | 0.52 | 0.45-0.58 | 0.605 | 1.90E-05 |
| PODXL | 0.55 | 0.48-0.61 | 0.114 | 3.34E-04 |
